# Investigating sex differences in T regulatory cells from cisgender and transgender healthy individuals and patients with autoimmune inflammatory disease: a cross-sectional study

**DOI:** 10.1016/S2665-9913(22)00198-9

**Published:** 2022-08-31

**Authors:** George A Robinson, Junjie Peng, Hannah Peckham, Gary Butler, Ines Pineda-Torra, Coziana Ciurtin, Elizabeth C Jury

**Affiliations:** a Centre for Rheumatology Research, Division of Medicine, University College London, London, UK; b Centre for Adolescent Rheumatology Versus Arthritis, Division of Medicine, University College London, London, UK; c Centre for Cardiometabolic and Vascular Science, Division of Medicine, University College London, London, UK; d Department of Paediatric and Adolescent Endocrinology, University College London Hospital and Great Ormond Street Institute of Child Health, University College London, London, UK; e Gender Identity Development Service, Tavistock and Portman NHS Foundation Trust, London, UK

## Abstract

**Background:**

Sexual dimorphisms, which vary depending on age group and pubertal status, have been described across both the innate and adaptive immune system. We explored the influence of sex hormones on immune phenotype in the context of adolescent health and autoimmunity.

**Methods:**

In this cross-sectional study, healthy, post-pubertal cisgender individuals (aged 16–25 years); healthy, pre-pubertal cisgender individuals (aged 6–11 years); transgender individuals (aged 18–19 years) undergoing gender-affirming treatment (testosterone in individuals assigned female sex at birth and oestradiol in individuals assigned male sex at birth); and post-pubertal cisgender individuals (aged 14–25 years) with juvenile-onset systemic lupus erythematosus (SLE) age-matched to cisgender individuals without juvenile-onset SLE were eligible for inclusion. Frequencies of 28 immune-cell subsets (including different T cell, B cell, and monocyte subsets) from each participant were measured in peripheral blood mononuclear cells by flow cytometry and analysed by balanced random forest machine learning. RNA-sequencing was used to compare sex and gender differences in regulatory T (Treg) cell phenotype between participants with juvenile-onset SLE, age-matched cis-gender participants without the disease, and age matched transgender individuals on gender-affirming sex hormone treatment. Differentially expressed genes were analysed by cluster and pathway analysis. Suppression assays assessed the anti-inflammatory function of Treg cells in vitro.

**Findings:**

Between Sept 5, 2012, and Nov 6, 2019, peripheral blood was collected from 39 individuals in the post-pubertal group (17 [44%] cisgender men, mean age 18·76 years [SD 2·66]; 22 [56%] cisgender women, mean age 18·59 years [2·81]), 14 children in the cisgender pre-pubertal group (seven [50%] cisgender boys, mean age 8·90 [1·66]; seven [50%] cisgender girls, mean age 8·40 [1·58]), ten people in the transgender group (five [50%] transgender men, mean age 18·20 years [0·47]; five [50%] transgender women, mean age 18·70 years [0·55]), and 35 people in the juvenile-onset SLE group (12 [34%] cisgender men, mean age 18·58 years [2·35]; 23 [66%] cisgender women, mean age 19·48 [3·08]). Statistically significantly elevated frequencies of Treg cells were one of the top immune-cell features differentiating young post-pubertal cisgender men from similarly aged cisgender women (p=0·0097). Treg cells from young cisgender men had a statistically significantly increased suppressive capacity in vitro compared with those from cisgender women and a distinct transcriptomic signature significantly enriched for genes in the PI3K–AKT signalling pathway. Gender-affirming sex hormones in transgender men and transgender women induced multiple statistically significant changes in the Treg-cell transcriptome, many of which enriched functional pathways that overlapped with those altered between cisgender men and cisgender women, highlighting a hormonal influence on Treg-cell function by gender. Finally, sex differences in Treg-cell frequency were absent and suppressive capacity was reversed in patients with juvenile-onset SLE, but sex differences in Treg-cell transcriptional signatures were significantly more pronounced in patients with juvenile-onset SLE compared with individuals without juvenile-onset SLE, suggesting that sex hormone signalling could be dysregulated in autoimmunity.

**Interpretation:**

Sex-chromosomes and hormones might drive changes in Treg-cell frequency and function. Young post-pubertal men have a more anti-inflammatory Treg-cell profile, which could explain inflammatory disease susceptibilities, and inform sex-tailored therapeutic strategies.

**Funding:**

Versus Arthritis, UK National Institute for Health Research University College London Hospital Biomedical Research Centre, Lupus UK, and The Rosetrees Trust.

**Research in context**

**Evidence before this study**

Sex differences in the innate and adaptive immune response result in differential responses to infection, vaccination, and susceptibility to autoimmune diseases. Studies have reported different sex specific observations by age group regarding inflammatory profiles and disease presentation. This is particularly relevant for juvenile-onset systemic lupus erythematosus (SLE), which predominantly effects young women and has a common onset at puberty. We searched PubMed, Web of Science, and Google Scholar for research articles published between Jan 1, 1990, and April 30, 2022, using the search terms “sex”, “gender”, “transgender”, “sex hormones”, “sex chromosomes”, “inflammation”, “immune response”, “(juvenile-onset) systemic lupus erythematosus”, and “autoimmunity”. We also searched for research articles published in the same time window in the top rated rheumatology, immunology, and endocrine-specific journals by impact factor. Published abstracts were excluded from the searches. The earliest referenced article was published in 2003. We found that investigating the role of sex and gender in immunological research is aiding the understanding of sexual dimorphisms in the immune system in both healthy individuals and in diseases with a sex bias. However, disparities across published results have emerged, probably because of differences in study age groups, pubertal status, gender consideration, and use of different animal models.

**Added value of this study**

To our knowledge, this study is the first to report an in-depth analysis of immune-cell phenotype comparing sex and gender differences between young (aged 14–25 years) post-pubertal cisgender men and cisgender women both with and without juvenile-onset SLE, and in transgender individuals undergoing gender-affirming sex hormone treatment who did not have juvenile-onset SLE. During adolescence, rapid changes in sex hormones drive changes in inflammatory responses and disease susceptibility. Therefore, this age range is important in the context of autoimmune research. We found unique changes in the immune profile, specifically in regulatory T cells, between cisgender men and cisgender women and sex hormone-associated transcriptomic profiles that overlap by gender, which could play a role in juvenile-onset SLE pathogenesis.

**Implications of all the available evidence**

The evidence from this study could be used to improve our understanding of sexual dimorphisms in immune responses by sex to improve sex-specific and gender-specific therapeutic strategies in health and disease and to understand sex differences in inflammatory responses, vaccine efficacy, and autoimmune disease susceptibility. We also highlight specific immune features and genes that could be targeted therapeutically or used for sex-specific diagnosis and investigation of juvenile-onset SLE pathogenic mechanisms.

## Introduction

Men and women differ in their inflammatory response to non-self and self antigens; consequently, they have different responses to vaccination and risks of infection.^1^ This has been highlighted by the COVID-19 pandemic^2^ and historically by the risk developing autoimmune diseases: men are generally more likely to have a more severe disease course following SARS-CoV-2 infection, whereas women represent about 80% of patients with autoimmunity.^1^, ^3^ Sex differences across the immune system, including fundamental differences in the frequency and activity of T-cell subsets, have been described by gender across ethnicities.^1^, ^4^ Generally, previous observations show higher CD4^+^ T-cell and lower CD8^+^ T-cell frequencies^4^, ^5^ and higher proinflammatory, cytotoxic, and antiviral T-cell responses in women compared with men. Of note, some sex differences in adaptive immunity are present throughout life whereas others manifest after the onset of puberty or post-reproductive senescence, implicating both genetic and sex hormonal influences.^7^

Systemic lupus erythematosus (SLE) is a complex autoimmune disorder characterised by loss of immune cell regulation and the presence of autoantibodies against nuclear and non-nuclear antigens, resulting in chronic inflammation and organ damage. Sex hormones have been hypothesised to be implicated in the cause of SLE due to significant sex bias (nine female patients to every one male patient) and frequent disease onset during childbearing age (15–44 years). Between 15% and 20% of all patients with SLE have juvenile-onset disease (onset before the patient reaches 18 years old). Juvenile-onset SLE often starts at puberty and has a more severe disease phenotype than adult onset disease.^8^ Together, this highlights a key role for sex hormones in susceptibility to juvenile-onset SLE.

Investigating the relationship between sex hormones and inflammation is important for understanding the cause of autoinflammatory diseases. Many clinical trials do not consider sex or gender in recruitment and outcome measures:^9^ historically less than 10% of immunological studies analysed data by sex.^10^ The absence of consideration of sex in research is particularly important for the study of autoimmune diseases, which are more prevalent in women.^3^ We aimed to investigate the influence of sex chromosomes and hormones in driving sexual dimorphisms in inflammatory profiles across different sex, gender, age, and disease status groups.

## Methods

### Study design and participants

In this cross-sectional study healthy, post-pubertal cisgender individuals (aged 16–25 years); healthy, pre-pubertal cisgender individuals (aged 6–11 years); transgender individuals (aged 18–19 years) undergoing gender-affirming treatment (testosterone in individuals assigned female sex at birth and oestradiol in individuals assigned male sex at birth); and post-pubertal cisgender individuals (aged 14–25 years) with juvenile-onset SLE age-matched to individuals without juvenile-onset were eligible for inclusion. Transgender individuals were contacted for participation via University College London Hospital's (London, UK) young people's Gender Identity Development Service liaison endocrine clinic, and participants with juvenile-onset SLE were contacted for inclusion via the Rheumatology Clinic at University College London Hospital. Patients were eligible to be included if they had juvenile-onset SLE according to the American College of Rheumatology revised classification criteria for lupus (1997) or the Systemic Lupus International Collaborating Clinics (SLICC) criteria (2012), and were diagnosed before the age of 18 years. For cisgender inviduals without juvenile-onset SLE, volunteers who did not have the disease or who had non-inflammatory, non-infective conditions (eg, referred for assessment of non-inflammatory musculoskeletal conditions) were recruited. Additional inclusion criteria for post-pubertal cohorts was a puberty Tanner stage of 4-5. Any patient who withheld consent or whose carer withheld consent for inclusion (as appropriate given patient's competence) and any patient who withdrew from the study were excluded from the analyses.

Ethics approval for the study was obtained from the London-Harrow Research Ethics Committee (REC11/LO/0330). Informed written consent was acquired from all participants (appendix 1 p 1).

### Procedures

Peripheral blood mononuclear cell (PMBC) samples were taken from cisgender individuals with and without juvenile-onset SLE and transgender individuals without juvenile-onset SLE. The samples were assessed for 28 immune-cell subsets (including different T-cell, B-cell, and monocyte subsets) by multiparameter flow cytometry, as described previously^11^ and in appendix 1 (pp 2–4).

To assess the suppressive capacity of regulatory T (Treg) cells in post-pubertal cisgender individuals with and without juvenile-onset SLE and transgender individuals without juvenile-onset SLE, fluorescence-activated cell sorting (FACS) was used to sort Treg cells, responder T (Tresp) cells, and monocytes for in-vitro suppression assays (appendix 1 p 5) using stored PBMCs from study participants collected during the research project. Treg-cell purity was confirmed by FOXP3 intracellular staining (appendix 1 p 5).

RNA sequencing was used to assess differentially expressed genes (DEGs) and gene ontology pathways between different sexes and genders with and without juvenile-onset SLE. Total RNA was isolated from FACS-sorted Treg cells (appendix 1 pp 5–6). Quality control analysis and sequencing was done by UCL Genomics (University College London, London, UK). Libraries were prepped with the NEB Low input kit (New England Biolabs, Ipswich, MA, USA) and were sequenced on the NovaSeq SP flow-cell (Illumina, San Diego, CA, USA) with a 100 base pair single read. Reads were demultiplexed and converted to fastq files with Illumina's bcl2fastq conversion software (version 2.19) and analysis was done by UCL Genomics. DEGs that were significantly different between cohorts, some of which were expanded using first-order protein–protein interaction networks, were analysed by hierarchical clustering (Pearsons), sparce partial least squares discriminant analysis, open target disease association analysis, and gene ontology pathway analysis to investigate differentially regulated immune functions and associations in Treg cells (appendix 1 p 6).

### Statistical analysis

For immunophenotyping, the analysis was exploratory based on limited sample availability, as a result of working with rare cohorts of young individuals and patients. For RNA sequencing, a sample estimation was carried out using a 90% power calculation (p<0·05) of data from a Treg-cell suppression assay (Treg-cell functional readout), using the proportion of Tresp cells suppressed in cisgender men versus cisgender women at a 1:1 Treg-cell to Tresp-cell ratio, yielding the required sample size of five participants per group (appendix 1 p 5). This indicated the number of samples needed to see functional transcriptomic changes in Treg cells by sex. Statistical analyses were done on the basis of the hypothesis that men and women have altered immune cell profiles, including Treg-cell phenotype, resulting in variation in inflammatory disease risk by sex and gender. Statistical analyses were done using GraphPad Prism (version 9). Demographic, clinical, immune frequency, DEG normalised count, and Treg-cell suppressive capacity measures data were tested for normal distribution. Normally distributed data were assessed with parametric tests; data that were not normally distributed data were assessed with non-parametric tests. For demographic, clinical, immune phenotype, gene expression, and Treg-cell suppression continuous data, unpaired t tests were used when comparing two groups and one-way ANOVA (Turkey's post-hoc test) tests were used when comparing more than two groups. For continuous data that were not normally distributed, Mann-Whitney tests were used when comparing two groups. For binary data Fisher's exact tests were used to compare two groups and χ^2^ tests were used when comparing more than two groups. p values less than 0.050 were considered significant unless mentioned otherwise. Multiple testing was accounted for using the false discovery rate adjustment for multiple comparisons Benjamini, Krieger, and Yekutieli method to calculate p values for immune phenotype data t tests. The balanced random forest machine-learning approach was used to assess the extent of the difference in the global immune phenotype between healthy, post-pubertal cisgender men and cisgender women and to highlight the key immune cell variables that were driving this difference. Receiver operator characteristic (ROC) analysis and 10-fold cross-validation were used to assess the balanced random forest model performance and classification accuracy, which was optimised and adjusted for age and ethnicity (appendix 1 p 6).

### Role of the funding source

The funding sources had no role in the study design, data collection, analysis, interpretation, or writing of the report.

## Results

Between Sept 5, 2012, and Nov 6, 2019, peripheral blood was collected from 39 individuals in the cisgender post-pubertal group (17 [44%] cisgender men, mean age 18·76 years [SD 2·66]; 22 [56%] cisgender women, mean age 18·59 years [2·81]; late puberty Tanner stage 4–5; appendix 1 p 1), 14 children in the pre-pubertal cisgender group (seven [50%] cisgender boys, mean age 8·90 years [1·66]; seven [50%] cisgender girls, mean age 8·40 years [1·58]; pre-puberty Tanner stage 1), ten individuals in the transgender group (five [50%] transgender men, mean age 18·20 years [0·47]; five [50%] transgender women, mean age 18·70 years [0·55]; mid to late puberty Tanner stage 2–5 at the time of puberty arrest), and 35 individuals in the juvenile-onset SLE group (12 [34%] cisgender men, mean age 18·58 years [2·35]; 23 [66%] cisgender women, mean age 19·48 [3·08]; late puberty Tanner stage 4–5). Participant characters are reported in the table.

**Figure 1:**
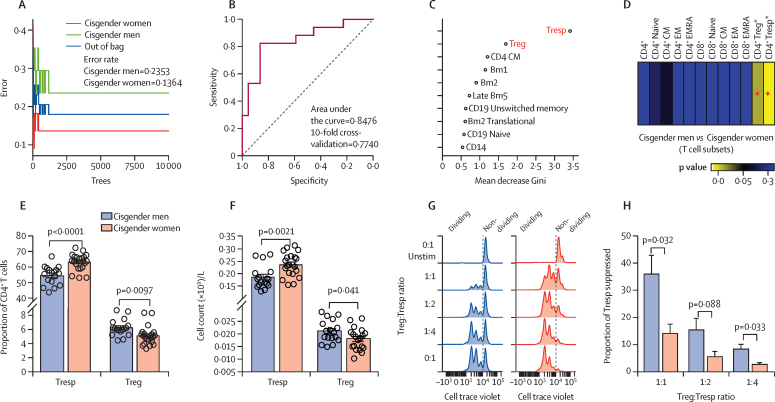
Comparison of immune-cell subsets in young-post pubertal cisgender men and women Comparison of 28 immune-cell subsets in 17 young, post-pubertal cisgender men versus 22 young, post-pubertal cisgender women using the balanced random forest model approach. (A) out of bag error rate of the balanced random forest was 0·1795 (82·05% accuracy). (B) Receiver operator characteristic curve analysis used to validate the model, providing an area under the curve of 0·8476 (84·76% accuracy; 82·61% sensitivity; and 81·25% specificity), with 10-fold cross-validation classification accuracy of 77·40%. (C) The top ten variables contributing to the balanced random forest: higher mean decrease in Gini score represents a higher importance of the variable. (D) Heatmap of p values comparing T-cell subset immunophenotyping in cisgender men versus cisgender women. Cumulative cell frequency (E) and absolute counts (F) Tresp-cell (CD4^+^CD25^–^CD127^+^) and Treg-cell (CD4^+^CD25^+^CD127^–^) frequencies; data are mean (SE). (G) Treg-cell mediated suppression of activated Tresp cells in four cisgender men and four cisgender women detected using cell trace violet and flow cytometry following 72 h activation using soluble anti-CD3 and anti-CD28 in the presence of monocytes. Treg-cell to Tresp-cell ratios of 1:1 1:2, 1:4, and 0:1 were assessed (appendix 1 p 5); each leftward peak represents a round of proliferation. (H) Suppressive capacity of Treg cells at varying Treg-cell to Tresp-cell ratios in cisgender men compared with cisgender women, calculated using the fold change of proportion of Tresp-cell proliferation with Treg cells (1:1, 1:2, and 1:4) compared with Tresp cells without Treg cells (0:1); data are mean (SE). CM=central memory. EM=effector memory. EMRA=effector memory re-expressing CD45RA. Treg=regulatory T. Tresp=responder T. *Significant p value following 10% false discovery rate adjustment for multiple comparisons.

Following optimisation and adjustment for age and ethnicity, ROC analysis of the balanced random forest model showed a strong classification accuracy (84·76%) and good model efficiency in discriminating cisgender men from cisgender women by immune phenotype (figure 1A, B). From this analysis, the predictive sensitivity was 82·61% and specificity was 82·25% for the BRF model, and the 10-times cross-validation classification accuracy held a steady measure of 77·40%. The top contributing immune-cell features segregating cisgender men from cisgender women were CD4^+^CD25^+^CD127^–^ Treg cells and CD4^+^CD25^–^CD127^+^ Tresp cells (figure 1C). This was confirmed by analysis of the frequency and absolute number of Treg cells—which were significantly increased (frequency p=0·0097; absolute number p=0·041)—and Tresp cells—which were significantly reduced (frequency p<0·0001; absolute number p=0·0021)—in cisgender men compared with cisgender women (figure 1D–F; appendix 1 p 7). This balance was reflected in the Treg-cell to Tresp-cell ratio, which was significantly higher in cisgender men compared with cisgender women (appendix 1 p 7). Of note, no significant differences in the frequency of other CD4^+^ and CD8^+^ T-cell subsets were seen (figure 1D).

Treg cells from young cisgender men had a significantly higher capacity to suppress activated Tresp cells in vitro compared with Treg cells from young post-pubertal cisgender women (figure 1G, H; appendix 1 p 5). Thus, Treg cells from young cisgender men and women are numerically and functionally distinct.

**Figure 2:**
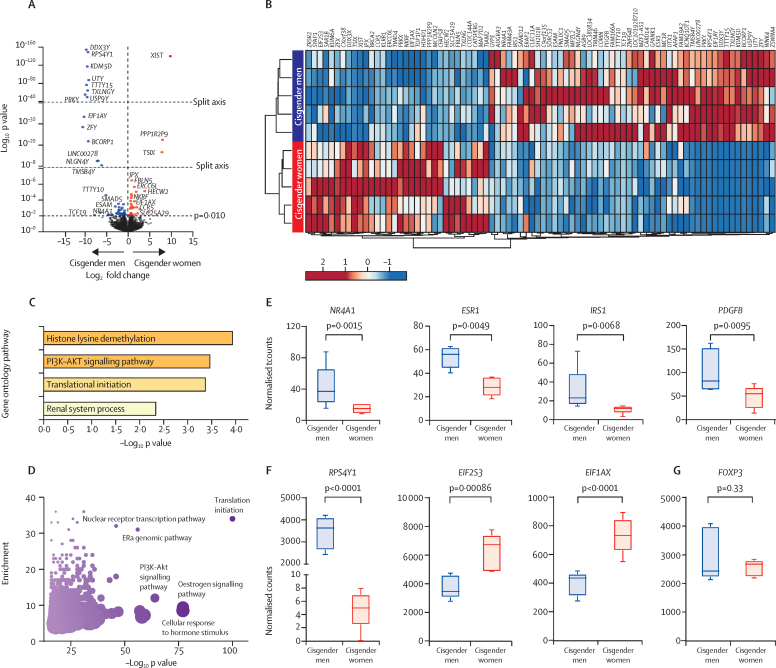
Comparison of Treg-cell transcriptomic profiles between young post-pubertal cisgender men and women FACS-sorted Treg cells (CD4^+^CD25^+^CD127^–^) from five young, post-pubertal cisgender men and five young, post-pubertal cisgender women were analysed by RNA-sequencing and whole genome expression was compared by sex. (A) Volcano plot displaying log_2_ fold changes and log_10_ p values of DEGs between cisgender men and women, where coloured points represent statistically significant DEGs below p value threshold of 0·01). (B) Hierarchical clustering heatmap (Clustvis, Pearson's) of normalised gene counts of statistically significantly altered DEGs between cisgender men and women. (C) Pathway analysis plot displaying the –log_10_ p values of enriched DEG pathway ontology terms of Treg cells between cisgender men and women using the 82 statistically significant DEGs. (D) Pathway analysis plot displaying the p value and enrichment ratios of enriched DEG pathway ontology terms of Treg cells between cisgender men and women using the extended gene list, incorporating both original seed genes and the genes from respective protein–protein interaction networks; the size of the points are relative to the number of genes contributing to that pathway. Box and whisker plots displaying Treg-cell gene expression by normalised counts of genes associated with the PI3K–AKT signalling (E), altered translation initiation pathways (F), and *FOXP3* expression (G); data are mean (SE). DEG=differentially expressed gene. ER=oestrogen receptor alpha. Treg=regulatory T. Tresp=responder T.

To investigate functional differences in Treg cells by sex, RNA sequencing, and DEGs from isolated Treg cells from young post-pubertal cisgender men and women were analysed. 82 genes were differentially regulated in Treg cells between cisgender men and women (figure 2A, B; appendix 2); there were 50 upregulated DEGs in cisgender men and 32 upregulated DEGs in cisgender women. The most significantly altered genes were located on the X or Y chromosomes: 16 (32%) DEGs in cisgender men and 18 (56%) DEGs in cisgender women (appendix 1 p 8). The other DEGs were expressed on other chromosomes typically shared equally by sex (appendix 1 p 8), suggesting both sex chromosomes and hormones could affect Treg-cell transcriptomes.

Gene ontology pathway enrichment analysis of the original 82 DEGs was done and validated using an extended gene list based on known protein–protein interactions (figure 2C, D; appendix 1 p 8). This highlighted and validated many significantly enriched functional pathways in Treg cells that differ by sex, including altered PI3K–AKT signalling, nuclear receptor transcription, translation initiation, oestrogen receptor signalling, and cellular response to hormone stimulus. Of note, several key node genes from the protein–protein interaction network (appendix 1 p 8) were exclusive to the PI3K–AKT signalling pathway and were increased in cisgender men compared with cisgender women, including *NR4A1*, *ESR1*, *IRS1*, and *PDGFB* (figure 2E; appendix 1 pp 9–10), supporting increased Treg-cell functionality in cisgender men. Other node genes from the extended network were associated with altered translation initiation pathways, including *EIF2S3*, *EIF1AX* (X-linked), and *RPS4Y1* (Y-linked); these were increased in a respective sex chromosome-dependent way (figure 2F; appendix 1 pp 9–10). No significant difference in Treg cell *FOXP3* expression was identified between cisgender men and cisgender women, despite its gene location on the X chromosome (figure 2G).

To explore the specific influence of sex hormones on the sexually dimorphic Treg-cell profiles, we used unique cohorts of cisgender, pre-pubertal children and young transgender individuals, who had their physiological puberty blocked followed by supplementation of gender-affirming sex hormones specific to their target gender (table; appendix 1 p 11).

**Table:** Demographic and clinical characteristics

		**Cisgender and transgender men**	**Cisgender and transgender women**	**p value**
**Cisgender post-puberty**				
Number of participants		17	22	..
Age (years)		18·76 (2·66)	18·59 (2·81)	0·85†
Ethnicity				
	White	8 (47%)	12 (55%)	0·75*
	Asian	6 (35%)	4 (18%)	0·14*
	Black	1 (6%)	2 (9%)	1·00*
	Other	2 (12%)	4 (18%)	0·68*
Tanner Stage at time of sample				
	Tanner stage 1	0	0	>1·00*
	Tanner stage 2–3	0	0	>1·00*
	Tanner stage 4–5	17 (100%)	22 (100%)	>1·00*
	Taking contraception (combined pill)	0	2 (9%)	0·50*
**Cisgender pre-puberty**				
Number of participants		7	7	..
Age (years)		8·90 (1·66)	8·40 (1·58)	0·68†
Ethnicity				
	White	4 (57%)	3 (43%)	>1·00*
	Asian	2 (29%)	3 (43%)	>1·00*
	Black	1 (14%)	1 (14%)	>1·00*
	Other	0	0	>1·00*
Tanner Stage at time of sample				
	Tanner stage 1	7 (100)	7 (100%)	>1·00*
	Tanner stage 2–3	0	0	>1·00*
	Tanner stage 4–5	0	0	>1·00*
**Transgender on gender affirming hormones**				
Number of participants		5	5	..
Age (years)		18·20 (0·47)	18·70 (0·55)	0·16†
Ethnicity				
	White	5 (100%)	5 (100)	>1·00*
	Asian	0	0	>1·00*
	Black	0	0	>1·00*
	Other	0	0	>1·00*
Tanner Stage at time of puberty block				
	Tanner stage 1	0	0	>1·00*
	Tanner stage 2–3	1 (20%)	2 (40%)	>1·00*
	Tanner stage 4–5	4 (80%)	3 (60%)	>1·00*
Time on gender affirming hormone treatment (months)		11·7 (11·08)	12 (7·16)	0·96†
**Cisgender post-puberty with juvenile-onset SLE**				
Number of participants		12	23	..
Age (years)		18·58 (2·35)	19·48 (3·08)	0·39†
Ethnicity				
	White	3 (25%)	10 (43%)	0·46*
	Asian	6 (50%)	7 (30%)	0·29*
	Black	3 (25%)	4 (17%)	0·67*
	Other	0	2 (9%)	0·54*
Tanner Stage at time of sample				
	Tanner stage 1	0	0	>1·00*
	Tanner stage 2–3	0	0	>1·00*
	Tanner stage 4–5	12 (100%)	23 (100%)	>1·00*
Clinical feature				
	Age at disease onset (years)	13 (5·37)	12 (3·00)	0·32†
	SLEDAI	2·67 (2·87)	3·21 (3·18)	0·62†
	Neurological involvement	1 (8%)	1 (4%)	1·00*
	Serositis involvement	2 (17%)	6 (26%)	0·31*
	Cutaneous involvement	10 (83%)	21 (91%)	0·69*
	Haematological involvement	5 (42%)	9 (39%)	1·00*
	Musculoskeletal involvement	9 (75%)	21 (91%)	0·31*
	Renal involvement	4 (33%)	5 (22%)	0·69*
	Erythrocyte sedimentation rate	14·71 (14·22)	25·26 (32·93)	0·23†
	dsDNA titre	53·5 (4·75–138·8)	9 (3–350)	0·46†
	C3	0·93 (0·29)	1·053 (0·35)	0·28†
	Lymphocyte count	1·74 (0·8)	1·68 (0·6)	0·78†
Treatment				
	Hydroxychloroquine	11 (92%)	20 (87%)	1·00*
	Mycophenolate mofetil	5 (42%)	13 (57%)	0·49*
	Prednisolone	5 (42%)	12 (52%)	0·72*
	Prednisolone dose (mg)	4·00 (6·34)	3·83 (4·57)	0·72*
	Vitamin D	2 (17%)	6 (26%)	0·19*
	Methotrexate	1 (8%)	2 (9%)	1·00*
	Azathioprine	4 (33%)	3 (13%)	0·20*

Data are mean (SD), n (%), or median (IQR). For transgender individuals, the Tanner stage was their most recent Tanner stage before puberty blocking therapy. Tanner stage 1 is classified as pre-puberty, Tanner stages 2–3 were classified as in the larche or gonadarche, and Tanner stages 4–5 were classified as post-puberty. For patients with juvenile-onset SLE, common clinical measures of disease are shown as well as treatments. Rituximab treatment was avoided in the cohort. C3=complement component 3. dsDNA=anti-double-stranded-DNA antibodies. SLE=systemic lupus erythematosus. SLEDAI=systemic lupus erythematosus disease activity index.

* p value obtained with Fisher's exact test.

† p value obtained with unpaired t test for normally distributed data and Mann-Whitney test for not normally distributed data.

**Figure 3:**
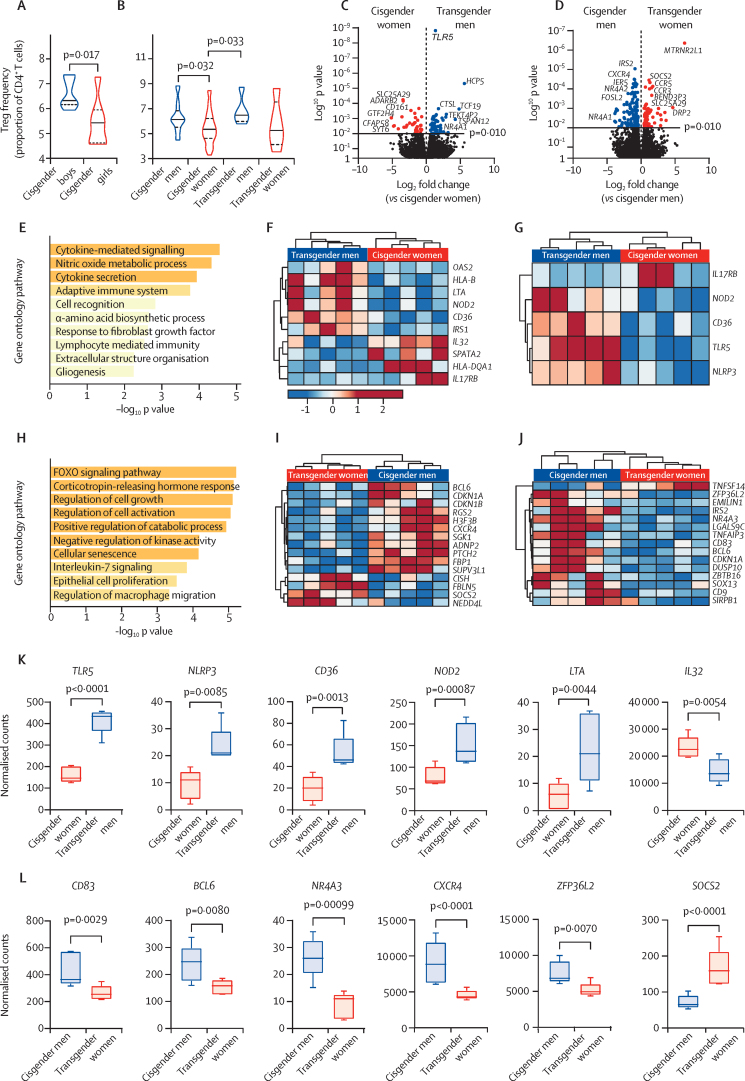
Assessment of Treg-cell functional gene expression and frequency in cisgender and transgender individuals Violin plots displaying circulating Treg-cell (CD4^+^CD25^+^CD127^–^) frequencies between seven pre-pubertal cisgender boys and seven cisgender girls, assessed by t test (A), and 17 post-pubertal cisgender men, 22 cisgender women, five transgender men, and five transgender women, assessed by one-way ANOVA (B); measured by flow cytometry; data are mean (SE). FACS-sorted Treg cells (CD4^+^CD25^+^CD127^–^) from five young, post-pubertal cisgender men and five cisgender women and five transgender men and five transgender women were analysed by RNA-sequencing and whole genome expression was compared by gender. Volcano plots displaying log_2_ fold changes and log_10_ p values of DEGs between transgender men and cisgender women (C) and transgender women and cisgender men (D): the red and blue points represent statistically significantly DEGs below p value threshold (p<0·01). Cluster significance –log_10_ p values of enriched DEG pathway ontology terms of Treg cells between transgender men and cisgender women (E). Hierarchical clustering heatmap (Clustvis, Pearson's) of normalised gene counts of DEGs found in the functional genetic pathway ontology terms in for transgender men versus cisgender women (F, G). Cluster significance –log_10_ p values of enriched DEG pathway ontology terms of Treg cells between transgender women versus cisgender men (H). Hierarchical clustering heatmap (Clustvis, Pearson's) of normalised gene counts of DEGs found in the functional genetic pathway ontology terms in for transgender women versus cisgender men (I, J). Box and whisker plots of Treg-cell gene expression by normalised counts of genes associated with the (K) cytokine signalling, and (L) cell growth and activation pathways; data are mean (SE). ANOVA=analysis of variance. DEGs=differentially expressed genes. FACS=fluorescence-activated cell sorting. Treg=regulatory T. Tresp=responder T.

Pre-pubertal cisgender boys had increased Treg-cell frequency compared with pre-pubertal cisgender girls (figure 3A; p=0·017). Furthermore, gender-affirming sex hormone therapy had an influence on Treg-cell frequencies: Treg cells were significantly increased in both cisgender men and transgender men compared with cisgender women, with a similar trend in cisgender men and transgender men compared with transgender women (figure 3B), suggesting that dimorphism in circulating Treg cell numbers might be driven by both sex hormones and underlying sex chromosomes.

In addition, large transcriptomic changes in Treg-cell gene expression were observed in both transgender men (treated with testosterone) compared with cisgender women (91 genes; figure 3C) and transgender women (treated with oestradiol) compared with cisgender men (235 genes; figure 3D; appendix 2), highlighting sex hormone-driven effects over chromosome effects. Pathway enrichment analysis highlighted key functional pathways in Treg cells that were altered by sex hormones in transgender individuals (figure 3E–J), including upregulated cytokine mediated signalling and cytokine secretion pathways in transgender men (figure 3E–G) and downregulated cell growth and activation pathways in transgender women (figure 3H–J). Many of these genes have individually been associated with Treg-cell function (figure 3K, L; appendix 1 pp 9–10).

**Figure 4:**
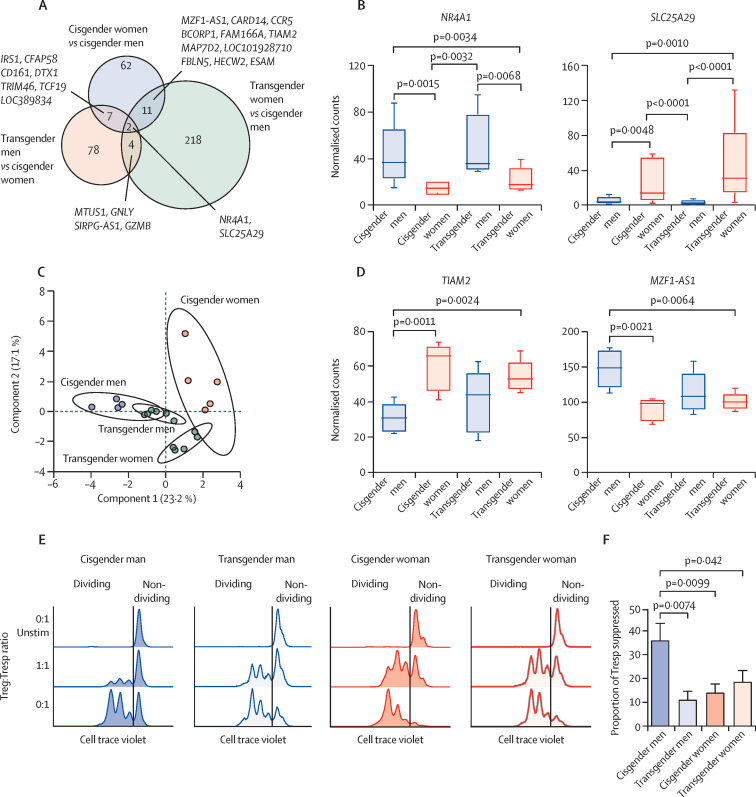
Sex and gender specific transcriptomic and functional changes in Treg cells Cohort details and gender-specific terminology descriptions are reported in appendix 1 (p 1). FACS-sorted Treg cells (CD4^+^CD25^+^CD127^–^) from five young, post pubertal cisgender men, five cisgender women, five transgender men, and five transgender women were analysed by RNA-sequencing and whole genome expression was compared by gender. (A) Overlap of DEGs (p<0·01) from Treg cells between different group comparisons: cisgender men versus cisgender women, transgender men versus cisgender women, and transgender women versus cisgender men. (B) Treg-cell gene expression by normalised counts of genes that overlapped in all gender comparisons; data were assessed by one-way ANOVA and are mean (SE). (C) Sparse partial least squares discriminant analysis plot clustering each gender group using normalised gene counts of the overlapping genes (n=24); individual distribution points and confidence ellipses (ovals) are plotted for each gender group. (D) Treg-cell gene expression by normalised counts of the top ranked loaded genes for component 1 (appendix 1 p 11); data were assessed by one-way ANOVA and are mean (SE). Treg-cell mediated suppression of activated Tresp cells. Treg cells, Tresp cells, and monocytes were isolated from four young, post-pubertal cisgender men, four cisgender women, three transgender men, and four transgender women. (E) Proliferation of Tresp cells at varying Treg-cell to Tresp-cell ratios in individuals of different genders. (F) Suppressive capacity of Treg cells at a Treg-cell to Tresp-cell ratio of 1:1 between different gender groups, calculated using the fold change of the percentage of Tresp-cell proliferation with Treg cells (1:1) compared with Tresp-cell proliferation without Treg cells (0:1); data were assessed with one-way ANOVA and are mean (SE; appendix 1 p 5). DEGs=differentially expressed genes. FACS=f luorescence-activated cell sorting. ANOVA=analysis of variance. Treg=regulatory T. Tresp=responder T. Unstim=Unstimulated.

The influence of sex hormones on Treg-cell gene expression was also compared between cisgender men and women and transgender men and women (figure 4A). Two genes were altered in all gender-unique comparisons: *NR4A1* and *SLC25A29* (figure 4B; appendix 1 pp 9–10), and 24 genes overlapped between at least two gender-unique comparisons (figure 4A; appendix 1 p 11). We were able to cluster gender-unique groups using hierarchical clustering (appendix 1 p 11) and sparse partial least squares discriminant analysis (figure 4C). The top ranked genes driving clustering in component 1 (highest contributing component) were *TIAM2* and *MZF1-AS1* (figure 4D; appendix 1 p 11). Of the 62 DEGs unique to the original cisgender analysis that did not overlap with transgender comparisons, 32 (52%) were X chromosome or Y chromosome linked (figure 4A). Of the remaining DEGs that did overlap with transgender comparisons, five genes (*BCORP1*, *MAP7D2*, *SLC25A29*, *NR4A1*, and *KLRB1*) were validated as being significantly altered between transgender men and transgender women, supporting a role of sex hormones in driving their expression (appendix 1 p 11; appendix 2).

Analysis of DEG lists using first-order protein–protein interaction extended network gene lists from cisgender and transgender comparisons revealed significant overlap of functional pathways associated with cell signalling by both secondary messengers and interleukins (appendix 1 p 12). Despite these functional transcriptomic changes, Treg cells from cisgender men were more suppressive in vitro than were Treg cells from all other individuals (figure 4E, F), suggesting that both sex chromosomes and hormones might play a role in the suppressive functions of Treg cells (appendix 1 p 13).

SLE is an autoimmune disease with a 90% female sex bias that is characterised by defective Treg function.^12^ Of note, a significant association was identified by open target analysis between the original 82 Treg-cell genes differentially expressed between cisgender men and women (figure 2A) and genes previously associated with SLE in public databases (p=0·020). This association was driven by ten genes (*ESR1*, *CCR5*, *TIMD4*, *DTX1*, *BRCA2*, *KDM6A*, *PDGFB*, *MC1R*, *NR4A1*, and *IRS1*; appendix 1 pp 9–10), and supports a role for these genes in sex specific SLE susceptibility. Of note, *KDM6A* is X chromosome linked, and *DTX1*, *IRS1*, *CCR5*, and *NR4A1* were regulated by testosterone, oestradiol, or both in transgender individuals on gender-affirming sex hormone treatment (figure 4A; appendix 1 p 11). Of the gender associated and SLE associated genes, *CCR5* and *ESR1* (increased in cisgender women), and *PDGFB* and *MC1R* (increased in cisgender men) are already established drug targets for HIV (*CCR5*); breast cancer, infertility, and obesity (*ESR1*); macular degeneration (*PDGFB*); and erythropoietic protoporphyria and kidney injury (*MC1R*; appendix 1 p 11).

**Figure 5:**
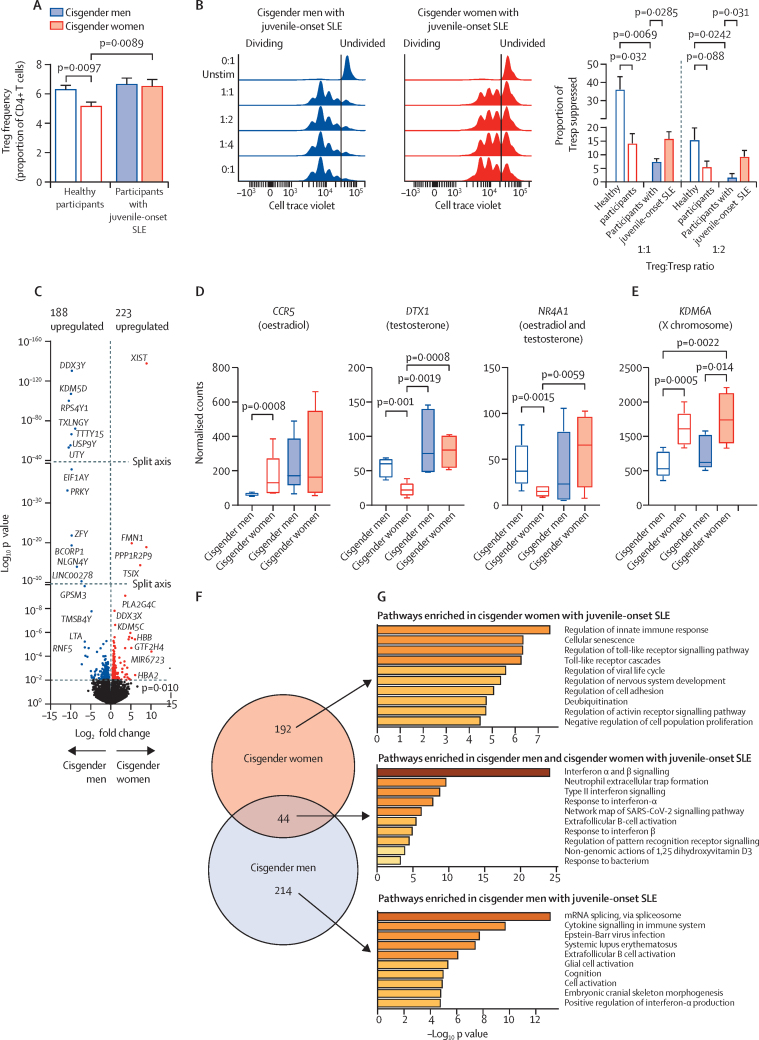
Sex differences in Treg cells in patients with juvenile-onset SLE (A) Cumulative cell frequency flow cytometry data of Treg cells (CD4^+^CD25^+^CD127^–^) comparing 17 young post-pubertal healthy cisgender men, 12 cisgender men with juvenile-onset SLE, 22 young post-pubertal healthy cisgender women, and 23 cisgender women with juvenile-onset SLE; data were assessed by t test and are mean (SE). (B) Treg cell-mediated suppression of activated Tresp cells in five cisgender men and five cisgender women with juvenile-onset SLE, and four cisgender men and four cisgender women without juvenile-onset SLE; data were assessed with t test and are mean (SE). (C) Log_2_ fold changes and log_10_ p values of DEGs between five cisgender men and five cisgender women with juvenile-onset SLE, coloured points represent statistically significantly upregulated DEGs below p value threshold (p<0·01). Treg-cell gene expression by normalised counts of hormone (gender) (D) or sex chromosome specific genes (E) that were significantly associated with SLE by open target analysis (five per group); data were assessed with one-way ANOVA and are mean (SE); open bars are participants without juvenile-onset SLE and closed bars are participants with juvenile-onset SLE. (F) Overlap of DEGs (p<0·01) from Treg cells comparing patients with juvenile-onset SLE with healthy participants, stratified by sex (cisgender men and cisgender women). (G) Pathway analysis bar charts of enriched pathway ontology terms from sex-unique or overlapping DEGs. ANOVA=analysis of variance. SLE=systemic lupus erythematosus. DEGs=differentially expressed genes. Treg=regulatory T. Tresp=responder T. Unstim=unstimulated.

Sex differences in Treg-cell frequencies were absent in juvenile-onset SLE, probably due to the significant increase in Treg-cell frequency in cisgender women with juvenile-onset SLE (figure 5A). Of note, there was no significant difference in clinical measures or treatments between cisgender men or cisgender women with juvenile-onset SLE (table 1), nor was there a significant effect of these features on Treg-cell frequencies in the combined juvenile-onset SLE cohort (appendix 1 p 13). This was observed despite multiple patients being treated with glucocorticoids (17 [49%] of 35 patients), hydroxychloroquine (31 [89%] patients), or methotrexate (three [9%]; appendix 1 p 13). Additionally, Treg cells from patients with juvenile-onset SLE were more suppressive in cisgender women compared with cisgender men, the opposite of that seen in individuals who do not have juvenile-onset SLE, probably due to the significant loss of Treg-cell suppressive function in cisgender men with juvenile-onset SLE (figure 5B).

When Treg-cell gene expression was compared between cisgender men with juvenile-onset SLE and cisgender women with juvenile-onset SLE, 411 DEGs were identified (figure 5C; appendix 2) compared with 82 sex specific genes in cisgender individuals who did not have juvenile-onset SLE (figure 2A; appendix 2). Of these genes, 223 were increased in cisgender women with juvenile-onset SLE compared with 188 in cisgender men with juvenile-onset SLE. Only 27 genes overlapped between the cisgender men and cisgender women in the juvenile-onset SLE and healthy post-pubertal groups (appendix 1 p 14); all 27 overlapping genes were X or Y chromosome linked. Gene ontology pathway analysis of the 384 genes altered by sex that were unique to the juvenile-onset SLE group showed significant enrichment of major histocompatibility complex class II antigen presentation, membrane trafficking, protein-DNA complex assembly, histone 3 lys 27 methylation, and ras protein signal transduction pathways (appendix 1 p 14). Furthermore, sex differences in Treg-cell functional pathways were more pronounced in patients with juvenile-onset SLE compared with those who did not have the disease (appendix 1 p 14).

Of note, sex differences in the expression of *CCR5*, *DTX1*, and *NR4A1* (genes that were significantly altered between cisgender men and cisgender women who did not have juvenile-onset SLE, were affected by gender affirming treatment hormones in transgender individuals and were associated with SLE by open target analysis), were absent in patients with juvenile-onset SLE (figure 5D). By contrast, sex differences in sex-chromosome linked genes (eg, KDM6A), that were associated with SLE by open target analysis, were maintained in patients with juvenile-onset SLE (figure 5E). In addition, comparing patients with juvenile-onset SLE with individuals who did not have the disease by sex showed very little overlap of DEGs between cisgender men and cisgender women, suggesting that sex hormones might play a differential role in juvenile-onset SLE pathogenesis and disease presentation by sex (figure 5F; appendix 1 p 13). This was validated by pathway enrichment analysis, in which genes unique to juvenile-onset SLE pathogenesis in cisgender men represented mRNA splicing and cytokine signalling, whereas those in cisgender women represented innate immune responses, cellular scenenscence, and toll-like receptor signalling (figure 5G). As expected, shared pathways included a strong upregulation of interferon signalling.

## Discussion

Our study identified key sex differences in Treg-cell phenotype and function between healthy individuals, which might explain differences in autoimmune disease susceptibilities and response to infection. Specifically, we identified that the global immune profile was altered by sex, with circulating Treg cells more numerous and suppressive in young post-pubertal cisgender men compared, with cisgender women; Treg cells had an altered transcriptomic profile between young cisgender men and cisgender women (beyond sex chromosomes) associated with increased secondary messenger signalling in cisgender men; sex hormones altered the Treg-cell frequency and transcriptomic functional profile between cisgender men and women, validated using transgender individuals undergoing gender-affirming sex hormone therapy; and differences in Treg frequency and function were absent or altered in an age-matched post-pubertal cisgender patients with juvenile-onset SLE. The information reported here might help us to understand immunopathological mechanisms of sexually dimorphic autoimmune disease development and contribute to basic understanding of immunology by sex and gender.

We showed that use of broad-spectrum immune phenotyping with machine learning can be used to classify sex differences with confidence. To our knowledge, no other studies have used machine learning to address innate and adaptive immunological differences between men and women, particularly surrounding prediction accuracy as a method to describe how different these sexual dimorphisms are. However, other studies, including our own, have addressed this from an autoimmunity standpoint, comparing individuals with and without specific diseases (eg, juvenile-onset SLE and juvenile idiopathic arthritis). These studies of similar immune landscapes found that a balanced random forest model had a strong classification accuracy: 90·9% (4·8% higher than this study) for classifying patients with juvenile-onset SLE,^11^ and 89·6% (6·1% higher than our study) for classifying patients with juvenile idiopathic arthritis^13^ from healthy controls. For identifying patients with juvenile-onset SLE, there was a diagnostic sensitivity of 89·6% (7·0% higher than this study) and specificity of 82·1% (0·2% lower than this study). This highlights that the extent of immune dimorphisms observed between healthy cisgender men and women are not too dissimilar to those observed between people who have and those who do not have juvenile-onset SLE, supporting our hypothesis of altered autoimmune risk by sex. Treg cells were one of the top contributing immune-cell features responsible for segregating cisgender men from cisgender women using machine learning. This supports previous observations of human sexual dimorphism in Treg-cell frequency across different post-pubertal age groups;^1^, ^14^ however, mouse studies of Treg cells by sex are contradictory, probably due use of disease models and examining organ-specific rather than peripheral blood Treg cells.^14^ We found that young post-pubertal cisgender men had increased Treg-cell frequency and suppressive function compared with cisgender women, suggesting a more anti-inflammatory circulating immune profile. These characteristics could contribute to the better response of women to vaccination and infection that has been observed (highlighted in the COVID-19 pandemic, in which men had an increased risk of mortality)^1^, ^2^ and the increased susceptibility of women to autoimmune disease compared with men.^3^ Despite these observations, multiple studies have reported a key role of oestradiol in supporting the expansion^15^, ^16^ and suppressive capabilities^17^, ^18^ of Treg cells. Treg-cell numbers have been shown to increase during the menstrual cycle when oestradiol concentrations are highest before ovulation,^19^ suggesting a Treg-cell response to sex hormones. By contrast, men who received gonadotropin-releasing hormone agonists, a puberty blocker which significantly reduces testosterone concentrations, have lower circulating Treg cells than do men who received placebo.^20^ As observed in cisgender men, we found that transgender men had increased Treg-cell frequencies following puberty blocking and early-stage gender-affirming testosterone administration (mean 12 months). Observed sex differences in Treg-cell frequencies in pre-pubertal children matched those seen in older participants post-puberty suggesting that sex hormones and chromosomes both play dominant roles in driving changes in Treg-cell frequency by sex. Women with Turner syndrome, characterised by the presence of one normal X chromosome and a missing or structurally abnormal second one, have an increased frequency of Treg cells compared with women with two intact X chromosomes, suggesting a more suppressive role of the X chromosome regarding Treg-cell proliferation.^21^ Despite these observations, no differences in Treg-cell frequencies have been reported in infants (who have sex chromosomes but very low concentrations of sex hormones) from birth to 1 year of age;^22^ therefore, the influence of the X chromosome on the number of circulating Treg cells might occur after the initial years of development in children.

Treg-cell *FOXP3* expression was not altered by sex in this study, despite its location on the X chromosome and previous reports of increased mRNA expression in cisgender men compared with women in an older cohort with a wider age range.^23^ Our data suggests that differences in Treg-cell function by sex might be maintained via different mechanisms in younger individuals. Transcriptomic analysis identified that the PI3K signalling pathway was increased in young cisgender men compared with young cisgender women in our study. PI3K signalling can support Treg-cell metabolism and function; however, overstimulation can reverse this in vitro.^24^ Inhibiting PI3K signalling in vivo results in reduced Treg-cell frequency and suppressive capacity,^25^ supporting a role for PI3K signalling in the increased suppressive capacity of Treg cells in young men compared with young women. Of note, secondary messenger signalling pathways were associated with hormone changes in transgender individuals by extended pathway analysis. A gene of interest from these pathways with increased expression in cisgender men and transgender men was *NR4A1* (and *NR4A3* in transgender men); these receptors are crucial for Treg-cell function,^26^ highlighting a therapeutic target to control autoimmune pathogenesis in women. Despite this, Treg cells in young cisgender men were more suppressive than those in transgender men and cisgender and in transgender women, suggesting that both sex hormones and sex chromosomes might play a role in driving increased suppresive function of Treg cells in cisgender men. Treg cells from women with Turner syndrome have impaired suppressive functions compared with women with two intact X chromosomes.^21^ A previous Treg-cell transcriptomics study combined a list of genes into a specific human Treg-cell signature, using previously published datasets, to produce a subsequent microarray test of 62 genes.^27^ Of these genes, we only identified a single gene, *CCR5*, that was significantly altered between cisgender men and cisgender women. *PECAM1* was the only altered gene in transgender men compared with cisgender women in our study. Our findings suggest that we might have identified multiple novel genes associated with Treg-cell function by sex. Pfoertner and colleagues^27^ analysed the combined data from six women and five men (aged 26–58 years) which might have substantially affected the results, as highlighted by our observations of sexual dimorphisms in Treg cells.

Although SLE is more common in females, sexual dimorphisms in SLE are important to consider because sex-specific clinical features have been observed in adults with SLE. Men are affected by more severe renal manifestations and higher end-stage renal disease risk, requiring increased monitoring in clinical practice.^28^ Of note, DEGs from Treg cells between young cisgender men and cisgender women were significantly associated with SLE, some of which were altered in transgender individuals. Treg cells have been implicated in SLE pathogenesis and disproportionate T helper 17-cell to Treg-cell ratios, resulting in a proinflammatory phenotype.^29^ However, these studies did not address sex differences and the populations included less than 7% men. It is also reported that women with SLE have increased plasma oestradiol and decreased testosterone concentration compared with women without SLE, which could account for differences in Treg-cell function; however, this study did not take into consideration the individual's stage of menstrual cycle.^23^ We showed that Treg cells were more numerous in patients with juvenile-onset SLE compared with individuals without juvenile-onset SLE in cisgender women, but not in men; therefore, Treg-cell frequency was not significantly different by sex in patients with juvenile-onset SLE. This increase in Treg-cell frequency in women with juvenile-onset SLE could represent an anti-inflammatory pathway aimed to counter the over-riding inflammation. We also showed that Treg cells from young cisgender women with juvenile-onset SLE had a significantly higher suppressive capacity compared with those from cisgender men with juvenile-onset SLE, the opposite of that seen in healthy individuals. However, this suppressive capacity of Treg cells from cisgender women with juvenile-onset SLE was still much lower than that observed in cisgender men and similar to that observed in cisgender women who did not have the disease. These findings suggest that the underlying function of Treg cells was not improved in cisgender women with juvenile-onset SLE, despite the increased number of cells. This could explain why anti-inflammatory feedback mechanisms are often not substantial enough to control disease; thus, therapies that promote Treg-cell function and number could still hold promise in juvenile-onset SLE for both sexes. We also identified strong sex differences in Treg-cell transcriptomic profiles in juvenile-onset SLE and highlighted both sex specific (mRNA splicing in cisgender men and immune regulation in cisgender women) and common (interferon signalling) pathogenic processes, which together might relate to sex differences in clinical outcomes for patients. Of note, we did not find an association between oestrogen-enriched and interferon-enriched pathways in the cohort of patients with juvenile-onset SLE in our study, which has been described in older patients (mean age 38·5 [SD 15·3]) with SLE.^33^ Finally, histone lysine demethylation was a key pathway altered between young cisgender men and cisgender women, supporting previous evidence surrounding the role of female-biased epigenetic alteration of T-cell function associated with SLE.^31^ Together, this supports a key role for Treg-cell targeted therapies in SLE and juvenile-onset SLE.^12^

Our study has several limitations. Difficulty in recruiting and taking larger volumes of blood from rare cohorts of young individuals, especially transgender individuals and children, limited our ability to assess the phenotype and function of Treg cells. Validation in larger cohorts of young individuals would be beneficial, but this is beyond the scope of this study. As highlighted by our study, it is also possible that Tresp cells could be important for the inflammatory balance between cisgender men and women, which would require additional phenotype and functional analyses. The increased suppressive capacity of Treg cells in cisgender men could also be due to increased proliferation of Tresp cells in cisgender women, or these cells not being as amenable to suppression compared with Tresp cells from cisgender men. We attempted to account for this by plotting the suppressive capacity of Treg cells as the fold change between Tresp-cell proliferation in the presence and absence of Treg cells, thus normalising the data for basal Tresp-cell proliferative capacity upon stimulation; however, this remains a study limitation. It was beyond the scope of the study to measure sex hormone concentrations in the serum of study participants; however, clinical monitoring aims to keep concentrations within physiological ranges. There was no follow-up of transgender individuals, which prevented us from observing the long-term effects of hormone therapy on Treg cells. Despite using a diverse healthy cohort for the immunophenotyping and accounting for ethnicity in analysis, the RNA-sequencing was done in White transgender individuals; thus, our findings will require validation in larger, more diverse cohorts. Although we have speculated on the functional implications on Treg cells of specific DEGs and pathways between sexes, the physiological relevance of these differences would need to be confirmed by mechanistic studies and analysis of proteins. The use of conventional flow cytometry limited the number of markers that we could measure for phenotyping. In future studies, it would be useful to investigate additional surface markers for Treg cells and Tresp cells to assess function and partition T cells into sublineages. A wider array of markers were used to this effect by Lambert and colleagues,^32^ who revealed important features that vary with autoimmune disease states and by age. Finally, the patients with juvenile-onset SLE were clinically heterogeneous and were on different treatments, presenting possible confounding effects on the immune system, especially considering that dsDNA has previously been shown to correlate negatively with Treg cells in adults with SLE^33^ and that immunosuppressive drugs, such as glucocorticoids, hydroxychloroquine, and methotrexate, could influence Treg-cell frequency and function.^34^, ^35^ Despite finding no difference in clinical measures or treatments between cisgender men and cisgender women with juvenile-onset SLE, or any association of these juvenile-onset SLE features with Treg frequency in our study, the potentially different treatment strategies are important to consider because treatments for the disease could explain the absence of differences in Treg-cell frequency in patients with juvenile-onset SLE. It is possible that this cohort—which includes patients with predominantly well controlled and inactive juvenile-onset SLE, with a high proportion of patients on hydroxychloroquine and several other treatments—might mask the potential effect of treatment and disease features on Treg-cell frequency and function by sex. Because juvenile-onset SLE is a severe disease frequently requiring timely commencement of therapy, recruitment of treatment-naive patients is not usually feasible.

To conclude, we have highlighted sexual dimorphisms in Treg-cell profiles and function by sex in young healthy individuals and patients with autoimmunity, showing differential influences of sex chromosomes and hormones using unique transgender cohorts. This information helps us to understand the mechanistic pathogenesis of autoimmune disease and the bias towards cisgender women. Our study will help inform the future consideration of sex as a biological variable in inflammatory research and clinical trials.

## Data sharing

Immune phenotype data can be found at Mendeley Data. RNA sequencing data can be found at ArrayExpress (Accession number: E-MTAB-11919) repositories. This data will be available from manuscript publication date.

## Declaration of interests

We declare no competing interests.
