# Supplementary material for: Investigating sex differences in T regulatory cells from cisgender and transgender healthy individuals and patients with autoimmune inflammatory disease: a cross-sectional study
Source: Lancet Rheumatol. 2022 Aug 31;4(10):e710–24. doi: 10.1016/S2665-9913(22)00198-9 (PMC9633330; doi:10.1016/S2665-9913(22)00198-9)
Supplement: Supplementary appendix 1 [file mmc1.pdf]

THE LANCET
Rheumatology

## Supplementary appendix 1

This appendix formed part of the original submission and has been peer reviewed. We post it as supplied by the authors.

Supplement to: Robinson GA, Peng J, Peckham H, et al. Investigating sex differences in T regulatory cells from cisgender and transgender healthy individuals and patients with autoimmune inflammatory disease: a cross-sectional study. *Lancet Rheumatol* 2022; published online Aug 31. https://doi.org/10.1016/S2665-9913(22)00198-9.

# Investigating sex differences in Tregs from cisgender and transgender healthy individuals and patients with autoimmune inflammatory disease: a cross-sectional study

## Supplemental Methods

### Patients and healthy cohorts/samples

Between Sept 5, 2012, and Nov 6, 2019, Peripheral blood was collected from 39 young, healthy, post-pubertal cisgender individuals (17 men, mean age (SD) 18.76 (2.66), 22 women, mean age (SD) 18.59 (2.81), all in late puberty Tanner stage 4-5), recruited from the local community at science outreach events (Table 1 and Supplemental Table 1). Blood was also collected from pre-pubertal healthy children recruited if blood was being taken for an unrelated clinical purpose (e.g. corrective surgery, screening), (Table 1) and from transgender individuals undergoing gender-affirming hormone treatment (testosterone in those born phenotypically female; trans-men, or oestradiol in those born phenotypically male; trans-women), recruited from UCLH young people's Gender Identity Development Service (GIDS) liaison endocrine clinic (Table 1 and Supplemental Table 1). Under current guidelines for NHS treatment of under 18s with gender incongruence, transgender individuals were treated with the gonadotropin-releasing hormone agonist triptorelin (GnRHa, "puberty blocker") for a minimum of 12 months prior to commencement of hormone treatment. All transgender individuals had completed or were nearing completion of physiological puberty of their birth registered sex prior to starting GnRHa treatment. Finally, peripheral blood was collected from 35 age matched young post-pubertal JSLE patients (12 cis-men, mean age (SD) 18.58 (2.35), 23 cis-women, mean age (SD) 19.48 (3.08), in late puberty Tanner stage 4-5), fulfilling The American College of Rheumatology (ACR) classification criteria for lupus (1997) (1) or the Systemic Lupus International Collaborating Clinics (SLICC) criteria (2012) (2), attending a young adult or adolescent rheumatology clinic at University College London Hospital (UCLH) (Table 1). Patients previously on B-cell depletion therapies or recently vaccinated were excluded. Informed written consent was acquired from all donors under the ethical approval reference: REC11/LO/0330. Questionnaires produced the self-rated Tanner puberty stage of all donors as well as their current use of contraception and any other relevant medication. All information was stored as anonymised data. Detailed demographic and any clinical characteristics, including treatment details, were recorded from NHS databases and questionnaires.

Gender terminology describing cohorts included in the study:
**Cis-woman:** a person who identifies as female and was **also** assigned female at birth. This may have been based on genitals and/or having two X chromosomes (XX).
**Cis-man:** a person who identifies as male and was **also** assigned male at birth. This may have been based on genitals and/or having one X and one Y chromosome (XY).
**Trans-woman:** a person who identifies as female but was assigned male at birth, on the basis of their genitals and/or having one X and one Y chromosome (XY). Sometimes known as *MTF; Male-to-female.*
**Trans-man:** a person who identifies as male but was assigned female at birth, on the basis of their genitals and/or having two X chromosomes (XX). Sometimes known as *FTM; Female-to-male.*

| Demographic: | Cis-men | Cis-women | Trans-men | Trans-woman | P value |
|---|---|---|---|---|---|
| Number | 5 | 5 | 5 | 5 | - |
| Age, mean (SD) | 23 (0.73) | 21.16 (2.41) | 18.20 (0.47) | 18.7 (0.55) | <0.0001‘‘ |
| **Ethnicity, number (%):** | | | | | |
| White | 2 (40) | 2 (40) | 5 (100) | 5 (100) | 0.036* |
| Asian | 3 (60) | 2 (40) | 0 (0) | 0 (0) | 0.066* |
| Black | 0 (0) | 1 (20) | 0 (0) | 0 (0) | 0.37* |
| **Tanner Stage at time of sample, number (%):** | | | | | |
| Tanner Stage 4-5 | 5 (100) | 5 (100) | - | - | >1.00* |
| **Tanner Stage at time of puberty block, number (%):** | | | | | |
| Tanner Stage 2-3 | - | - | 1 (20) | 2 (40) | >1.00* |
| Tanner Stage 4-5 | - | - | 4 (80) | 3 (60) | >1.00* |
| Time on cross-sex-hormone treatment (months), mean (SD) | - | - | 11.7 (11.08) | 12 (7.16) | 0.96$^{+}$ |

**Supplemental Table 1: Demographic and pubertal status comparison between cis- and trans-gender men and women analysed for RNA-sequencing**

*Fisher's exact (two groups) or chi-square (four groups) test, ‘‘one-way ANOVA, or $^{+}$unpaired t test was used. For transgender individuals, the Tanner stage was their most recent Tanner stage prior to puberty blocking therapy. Tanner stages 4-5 are classified as post-puberty.

## Flow cytometry

*Immune phenotyping:* As described previously (3), peripheral blood mononuclear cells (PBMCs; $1 \times 10^6$) were stained with fixable blue dead cell stain (ThermoFisher) and a T-cell or antigen presenting cell antibody panel (Supplemental Table 2 and 3 for cell types antibodies used) and followed by subsequent washes and fixation in 2% paraformaldehyde. Data acquisition was on a BD LSRFORTESSA X-20 flow cytometer (BD Biosciences; $1 \times 10^6$–$2 \times 10^6$ cells per sample), and FlowJo Analysis Software (TreeStar) was used to assess frequencies of 28 immune cell subsets (Supplemental Table 2 for cell types and Supplemental Figure 1 for gating strategies). Cytometer Setup and Tracking (BD Biosciences) beads were run to assess cytometer performance. Application settings were created and applied to panel templates before fluorochrome compensation to ensure that all immunophenotyping data were comparable over time. Absolute cell counts were calculated by multiplying the percentage of the population of interest in the total lymphocyte count by the average UCLH reference guidelines for healthy donor lymphocyte count ($1.225 \times 10^9$ cells/litre).

*Cell sorting:* PBMCs were washed in MACS buffer (PBS (Sigma), 2% FBS (Labtech) and 1mM EDTA (Sigma) and stained for 30 minutes with anti-human CD4-BUV395 (BD Biosciences), CD25-PE-Dazzle594 (Biolegend), CD127-BV711 (Biolegend) and CD14-APC (Biolegend) and sorted using a BD FACS Aria cell sorter into collection media (1xPBS, 20% FBS) (Supplemental Figure 2A for sort gating strategy). Purity checks were completed on sorted cells (Supplemental Figure 2B).

| | Cell type | Markers |
|---|---|---|
| **T-cells** | CD4 T-cell | CD3+, CD4+ |
| | CD8 T-cell | CD3+, CD8+ |
| | Naïve CD4 T-cell | CD4+, CD27+, CD45RA+ |
| | Central memory (CM) CD4 T-cell | CD4+, CD27+, CD45RA- |
| | Effector memory (EM) CD4 T-cell | CD4+, CD27-, CD45RA- |
| | Effector memory (EM) RA CD4 T-cell | CD4+, CD27-, CD45RA+ |
| | Naïve CD8 T-cell | CD8+, CD27+, CD45RA+ |
| | Central memory (CM) CD8 T-cell | CD8+, CD27+, CD45RA- |
| | Effector memory (EM) CD8 T-cell | CD8+, CD27-, CD45RA- |
| | Effector memory (EM) RA CD8 T-cell | CD8+, CD27-, CD45RA+ |
| | Regulatory T-cell (Treg) | CD4+, CD25+, CD127- |
| | Tresponder T-cell (Tresp) | CD4+, CD25-, CD127+ |
| | Invariant natural killer T- (iNKT) cells | CD3+, iTCR+ |
| **B-cells** | B-cells | CD19+ |
| | Bm1 (naïve) | IgD+, CD38- |
| | Bm2 (mature) | IgD+, CD38+ |
| | Bm2' (Transitional) | IgD+, CD38++ |
| | Bm3-4 (plasmablasts) | IgD-, CD38++ |
| | Early Bm5 (early memory) | IgD-, CD38+ |
| | Late Bm5 (late memory) | IgD-, CD38- |
| | Naïve | IgD+, CD27- |
| | Unswitched memory | IgD+, CD27+ |
| | Switched memory | IgD-, CD27+ |
| **Monocytes** | Monocytes | CD14+ |
| | Classical | CD14+, CD16- |
| | Non-classical | CD14+, CD16+ |
| | Intermediate | CD14-, CD16+ |
| **PDC** | Plasmacytoid dendritic cell (PDC) | CD303+ |

**Supplemental Table 2. Markers used to identify cell types for immunophenotyping by flow cytometry.**
List of markers used to define all 28 immune cell subsets (T-cells, B-cells, monocytes and PDCs) used in the paper analysis. These markers were targeted by antibodies described in Supplemental Table 3.

| Fluorochrome | T-cells | Company | Clone | APCs | Company | Clone |
|---|---|---|---|---|---|---|
| **UV 350⁄450** | Blue Dead Stain | ThermoFisher | N/A | Fixable Blue Dead Stain | ThermoFisher | N/A |
| **BUV395** | CD4 | BD | SK3 | CD19 | BD | SJ25C1 |
| **AF700** | CD27 | Biolegend | M-T271 | - | - | - |
| **BV421** | CD8a | Biolegend | RPA-T8 | CD38 | Biolegend | HB-7 |
| **BV510** | - | - | - | IgD | Biolegend | IA6-2 |
| **BV711** | CD127 | Biolegend | A019D5 | CD14 | Biolegend | M5E2 |
| **BV785** | CD3 | Biolegend | OKT3 | HLA-DR | Biolegend | L243 |
| **PERCP Cy5.5** | - | - | - | CD303 | Biolegend | 201A |
| **PE** | TCR Vα24-Jα18 | Biolegend | 6B11 | - | - | - |
| **PE-Dazzle594** | CD25 | Biolegend | M-A251 | CD16 | Biolegend | 3G8 |
| **PE-CY7** | CD45RA | Biolegend | HI100 | CD27 | Biolegend | M-T271 |

**Supplemental Table 3. Table of antibodies used for flow cytometry**
Antibodies used for immunophenotyping by flow cytometry. Two panels were developed, one for T-cells and one for antigen presenting cells (APCs). Target marker, conjugated fluorochrome, company and clone are displayed.

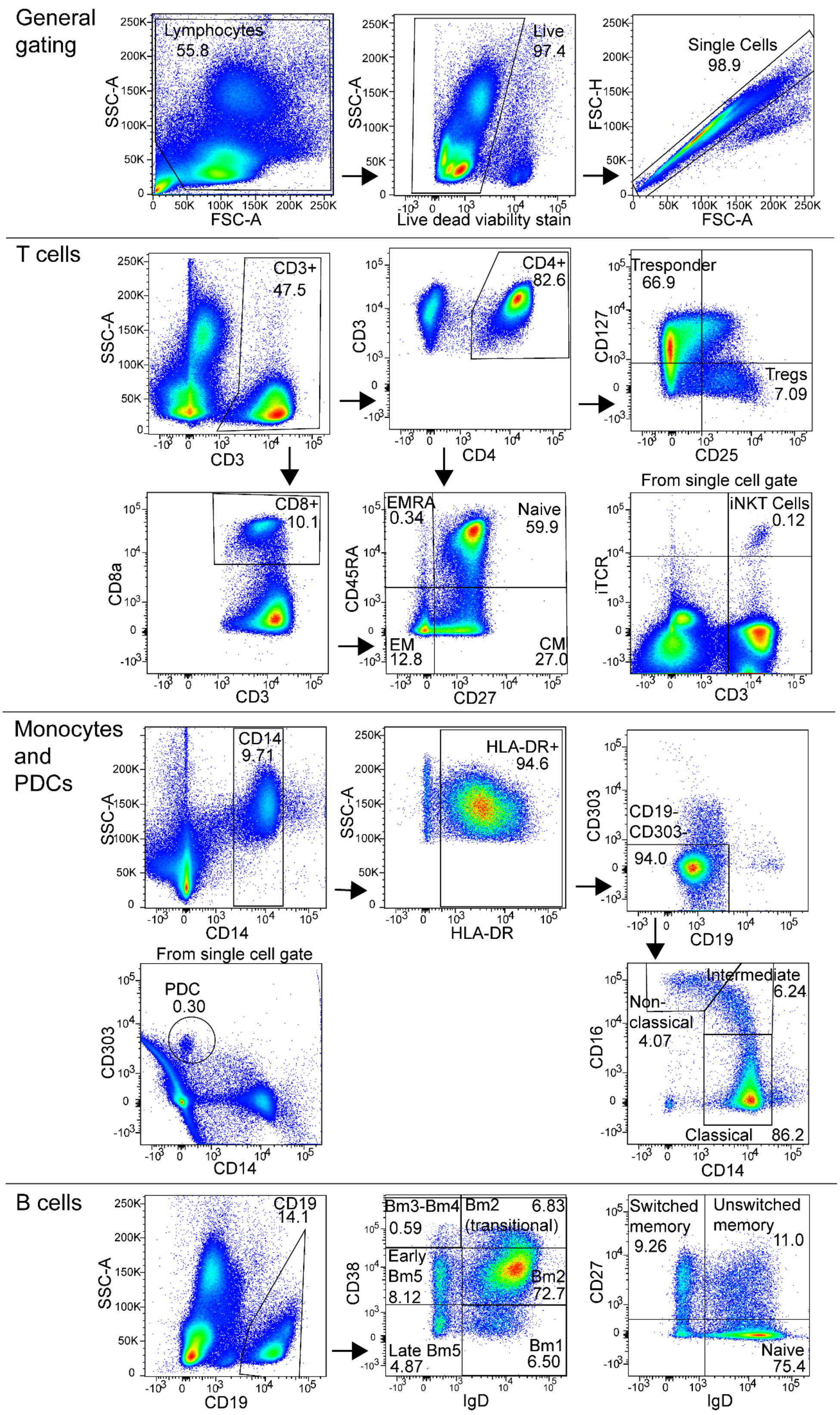


**Supplemental Figure 1: Gating strategies for flow cytometry immunophenotyping**

Representative gating strategies from a healthy donor used to identify T-cells, B-cells, Monocytes and PDC subsets. PBMC's were stained with antibodies outlined in methods. Samples were analysed using Fortessa X20 flow cytometer and Flowjo software. Labels represent the cell population within the gate. Regulatory T-cells (Tregs), central memory (CM), effector memory (EM). Representative Foxp3 percentage expression is shown in Supplemental Figure 4B where PBMC's were stained separately with CD4, CD127, CD25 and intracellularly stained with Foxp3 to validate the purity of Tregs taken from a CD25+CD127- gate.

**Suppression assays**

FACS sorted Tregs, Tresp and monocytes were used for suppression assays. Treg purity was confirmed by FOXP3 intracellular staining (Supplemental Figure 2B). Tresp cells ($CD25^-CD127^+$) were labelled for 20 minutes at room temperature with Cell Trace Violet (CTV, thermofisher) in PBS and cultured at a density of $8x10^4$ cells/well in RPMI 1640 medium (Thermofisher) supplemented with 10% FBS (ThermoFisher), Penicillin (100 IU/ml) and Streptomycin (100 µg/ml) (Gibco) with 1ug/mL soluble anti-CD3 (OKT3 mAb) (ebioscience) and 1ug/ml soluble anti-CD28 (ebioscience) in a 96 well flat bottom plate. Cells were co-cultured with unlabelled Treg cells at equal and descending density as well as monocytes at set density of $5x10^4$. Proliferating cells expressing CTV were fixed in PFA and quantified by flow cytometry after 72 hours (BD LSRFORTESSA X-20 flow cytometer (BD Biosciences, San Jose, CA, USA) and analysis with FlowJo software (TreeStar, San Jose, CA, USA) was used. Additional surface staining of CD4 was carried out to ensure monocytes were excluded from the analysis (Supplemental Figure 3 for gating strategy). The suppressive capacity of Tregs at varying Treg:Tresp ratios in men compared to women was calculated using the fold change of Tresp % proliferation with Tregs (1:1, 1:2, 1:4) compared to Tresp without Tregs (0:1).

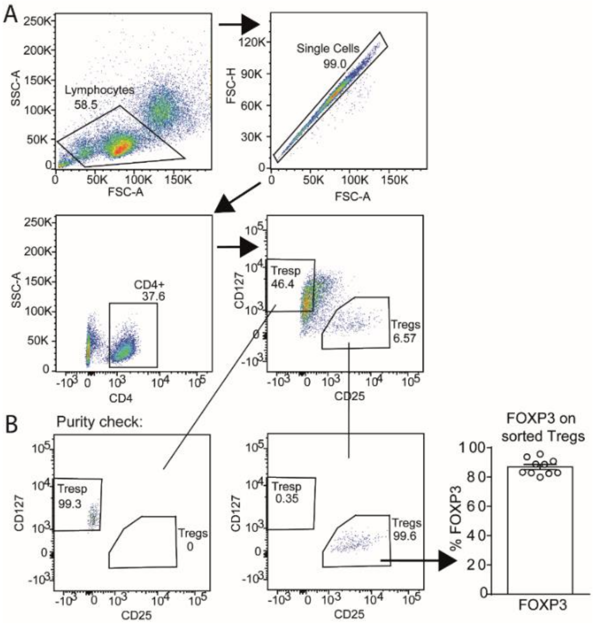


**Supplemental Figure 2: Gating strategy for Tregs and Tresp cell sorting and FOXP3 purity checks.**
**(A)** PBMCs were surface stained with CD4, CD25 and CD127 and sorted by a FACS Aria cell sorter. **(B)** A small aliquot of Tregs were tested for purity each time through intracellular staining of FOXP3. Tregs and Tresp cells were then used for suppression assays or qPCR.

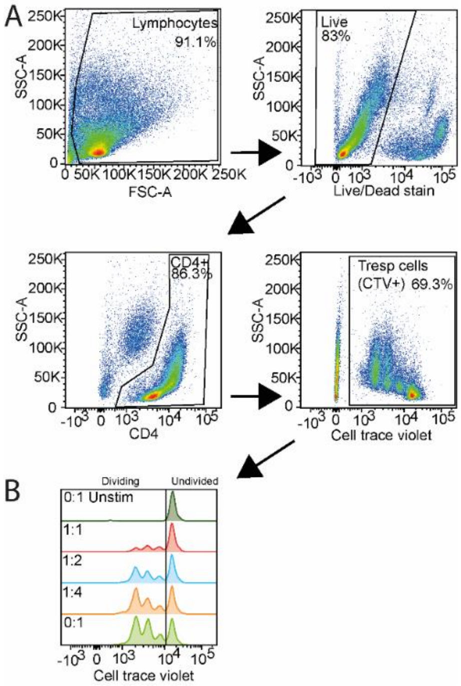


**Supplemental Figure 3: Gating strategy for Treg suppression assay analysis**
**(A)** Gating strategy for identification of Tresp (cell trace violet (CTV) positive) cells. **(B)** Representative plot of Tresp CTV expression through rounds of proliferation at varying ratios of Treg:Tresp cells. Undivided and dividing cells are labelled.

**RNA sequencing**

For RNA sequencing, a sample estimation was carried out using a power calculation (power=90%, $p<0.05$) of data from a Treg suppression assay (Treg functional readout), using the percentage of Tresp cells suppressed in men vs women at 1:1 Treg:Tresp ratio, yielding the required sample size of n=5/group (Supplemental Table 1). This indicated the number of samples needed to see functional transcriptomic changes in Tregs by sex.

*RNA extraction:* Total RNA was isolated from FACS sorted Tregs using the PicoPure RNA isolation kit (Applied Biosystems). RNA integrity was confirmed using Agilent's 2200 Tapestation. UCL Genomics (London, UK) performed cDNA library preparation using the NEB RNA Ultra II Directional assay with Poly(A) mRNA workflow (p/n E7760) according to manufacturer's instructions. Briefly, mRNA was isolated from total RNA using Oligo dT beads to pull down polyadenylated transcripts. The purified mRNA was fragmented using chemical hydrolysis (heat and divalent metal cation) and primed with random hexamers. Strand-specific first strand cDNA was generated and "A-tailed" at the 3' end. Full length xGen adaptors (IDT), containing unique 8bp dual sample specific indexes, a unique molecular identifier and a T overhang are ligated to the A-Tailed cDNA. Successfully ligated cDNA molecules were then enriched with limited cycle PCR.

*Sequencing:* Sequencing and quality control analysis was performed by UCL Genomics. Libraries were prepped with the NEB Low input kit and they were sequenced on the NovaSeq SP flow-cell (Illumina) with a 100bp single read.

*Analysis:* Reads were demultiplexed and converted to fastq files using Illumina's bcl2fastq Conversion Software v2.19 and analysis was performed by UCL Genomics. Briefly, the statistical analysis process included data normalization, graphical exploration of raw and normalized data, test for differential expression for each feature between the groups, raw p-value adjustment and export of lists of features having a significant differential expression between the groups. The analysis was performed using the R software R Core Team (4), Bioconductor (5) packages including DESeq2 (6, 7) and the SARTools package developed at PF2 - Institut Pasteur. Normalization and differential analysis are carried out according to the DESeq2 model and package. This report comes with additional tab-delimited text files that contain lists of differentially expressed features. Volcano plots of DEGs were produced using GraphPad Prism-9. Open targets (https://www.opentargets.org/) was used for disease association and drug target analysis using lists of DEGs. Pathway enrichment, network analysis, comparative heat maps and circos plots were performed using the web-based portal Metascape (http://metascape.org) on a list of statistically significant ($p<0.01$) differentially expressed genes (DEGs) in Tregs from healthy individuals with different genders. This provided a comprehensive gene list annotation curated via KEGG, Reactome and gene ontology databases. hierarchically clustered heatmaps were produced using ClustVis (http://biit.cs.ut.ee/clustvis/) and sPLS-DA and loadings plots produced using metaboanalyst (https://www.metaboanalyst.ca/). Venn diagrams were generated with BioVenn (http://www.biovenn.nl/index.php). Protein-protein interaction (PPI) networks were produced using NetworkAnalyst (https://www.networkanalyst.ca/), using the first-order minimum network tool, for the essential interactome of seed genes (DEGs, $p<0.01$) through the Search Tool for the Retrieval of Interacting Genes (STRING) database (8). This utilises a confidence score scheme indicating the estimated likelihood a given interaction is biologically plausible. A filter was applied to exclude interactions with a score <800 to reduce the likelihood of including false positives and filter low-scoring edges attributable to noise.

**Machine Learning**

The balanced random forest (BRF) approach was used with the randomForest package in R (9). A balanced random forest (BRF) is an ensemble machine learning algorithm for classification, consisting of numerous decisions trees which can increase model accuracy without the risk of model overfitting (10) which is often a problem when analysing data with small sample size such as in this study. In addition, the predictive performance of the BRF model can be estimated and assessed by 10-fold cross validation which mitigates the need for independent validation, giving an advantage when investigating rare cohorts (11), as appropriate in this study and as shown previously by our group (12). As the original sample set had an unbalanced men:women (17:22) ratio, the balanced method was applied in the bootstrap dataset construction, comparing equal numbers of men and women at each split. A parameter-tuning test was performed to maximise the model performance and 10,000 decision trees were used for model construction to ensure the reliable predictive performance of the model. Samples that were not included in the bootstrap dataset were termed the Out-of-Bag (OOB) dataset and were used to validate the model performance. Demographic factors (age and ethnicity) were included into the BRF model for adjustment purposes. For model performance evaluation, the receiver operator characteristic (ROC) plot and the area under the curve (AUC) of each model was computed with the pROC package in R (4). Validation was by 10-fold cross validation was applied with the caret package in R (13).

## Supplemental Results

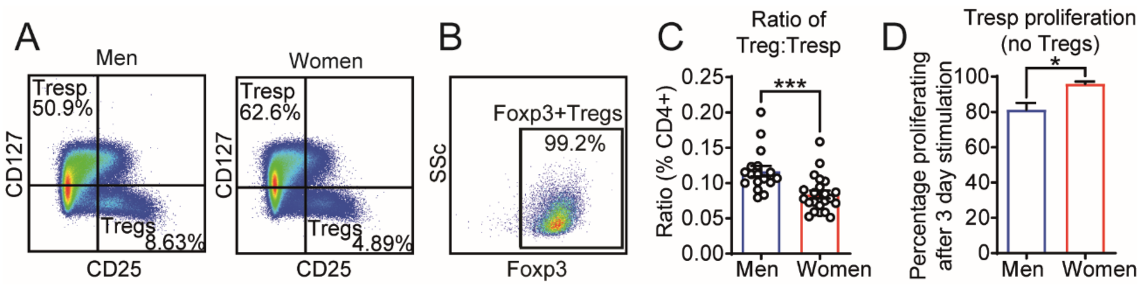


**Supplemental Figure 4: Representative plots of Tregs and Tresp in men and women**
**(A)** Representative plots of responder (Tresp, CD4+CD25-CD127+) and regulatory (Treg, CD4+CD25+CD127-) T-cell frequencies comparing young post-pubertal men and women from Figure 1. **(B)** Representative plot of FOXP3 expression validation in Tregs gated as per (A) and Supplemental Figure 1. **(C)** Treg:Tresp ratio (frequency of CD4+ T-cells) between men (n=17) and women (n=22). Mean+SE. t test, ***=p<0.001. **(D)** Percentage of proliferating Tresp cells without the presence of Tregs between men (n=4) and women (n=4) detected using cell trace violet (CTV) and flow cytometry following 72hour activation using soluble anti-CD3/28 in the presence of monocytes. Mean+SE. t test, *=p<0.05.

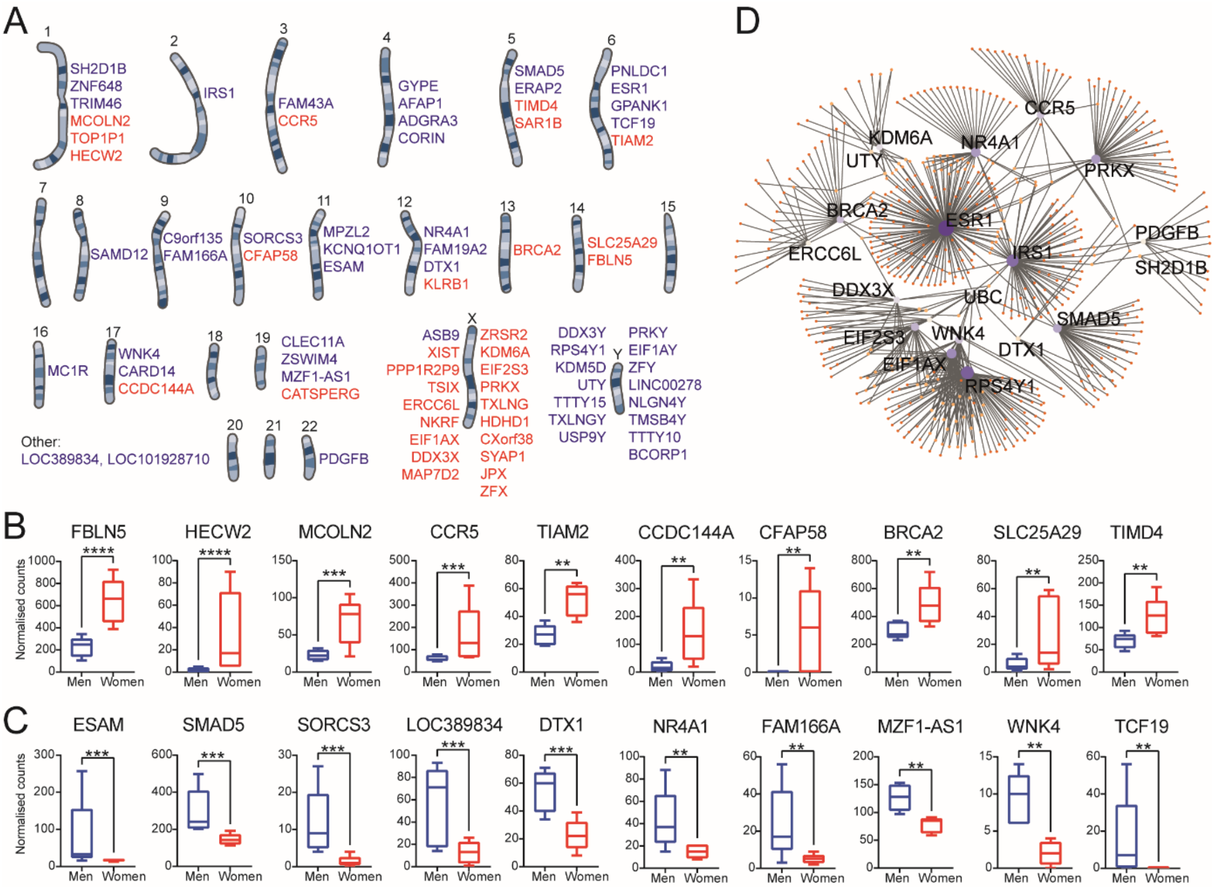

**Supplemental Figure 5: Chromosomal location and protein-protein interaction network of DEGs between men and women**

**(A)** Chromosomal location of all 82 significantly altered DEGs between men and women from Figure 1A-B, including genes upregulated in men (blue) and women (red). **(B-C)** Box and whisker plots displaying Treg gene expression by normalised counts of DEGs that represent the top 10 most significantly altered genes between men and women, those upregulated in **(B)** men or **(C)** women, that are not expressed on the X or Y chromosome. Mean+SE. t test, **=p<0.01, ***=p<0.001, ****=p<0.0001. Numerical p values can be found in the Supplemental Data Table. Protein-protein interaction (PPI) networks (Network Analyst) were developed for respective DEGs (seed genes, n=82) from statistically significantly altered DEGs between men and women from Figure 1A-B and were extended to a first-order network to encompass proteins that directly interact with the respective seed genes, identifying functional PPIs. **(D)** Graphical representation of the first-order network, encompassing proteins (orange nodes) that directly interact (connecting lines) with the respective seed genes (purple nodes). Node sizes are relative to the number of PPIs for respective genes, where genes with the most respective PPIs are labelled.

| Gene ID | Protein | Associated gender | Highlights in this study | Previously identified role in Tregs | References |
|---|---|---|---|---|---|
| NR4A1 | Nuclear receptor subfamily 4 group A member 1 | Cis-Men<br>Trans-men | – Enriched the PI3K/AKT signalling pathway<br>– Top 10 genes upregulated in cis-men (non-X/Y)<br>– Genes altered in all gender-unique comparisons<br>– Genes associated with sex/gender and SLE by open target analysis | – Essential for thymic Treg development and immune homeostasis<br>– Directly controls a genetic program indispensable for Treg maintenance, function, and immune suppression<br>– Regulates T-cell tolerance to suppress autoimmunity via Tregs<br>– Modulates Treg differentiation and clonal deletion | (14-17) |
| ESR1 (NR3A1) | Estrogen receptor 1 (nuclear receptor subfamily 3, group A, member 1) | Cis-Men | – Enriched the PI3K/AKT signalling pathway<br>– Genes associated with sex/gender and SLE by open target analysis | – Stimulation promotes Treg FOXP3 expression, expansion and suppressive activity | (18-20) |
| IRS1 | Insulin receptor substrate 1 | Cis-Men<br>Trans-Men | – Enriched the PI3K/AKT signalling pathway<br>– Genes associated with sex/gender and SLE by open target analysis | - | - |
| PDGFB | Platelet derived growth factor subunit B | Cis-Men | – Enriched the PI3K/AKT signalling pathway<br>– Genes associated with sex/gender and SLE by open target analysis | – Required for stimulatory effect of Tregs on fibroblasts | (21) |
| EIF2S3 | Eukaryotic translation initiation factor 2 subunit gamma | Cis-Women | – Enriched the translation initiation pathway | - | - |
| EIF1AX | Eukaryotic translation initiation factor 1A X-linked | Cis-Women | – Enriched the translation initiation pathway | - | - |
| RPS4Y1 | Ribosomal protein S4 Y-linked 1 | Cis-Men | – Enriched the translation initiation pathway | - | - |
| TLR5 | Toll Like Receptor 5 | Trans-Men | – Upregulated the cytokine-mediated signaling pathway in trans-men | – Increases Treg suppressive capacity | (22-24) |
| NLRP3 | NLR Family Pyrin Domain Containing 3 | Trans-Men | – Upregulated the cytokine-mediated signaling pathway in trans-men | – Negatively regulates Treg differentiation | (25) |
| CD36 | CD36 Molecule | Trans-Men | – Upregulated the cytokine-mediated signaling pathway in trans-men | – Supports Treg survival and function in tumours | (26) |
| NOD2 | Nucleotide-binding oligomerization domain-containing protein 2 | Trans-Men | – Upregulated the cytokine-mediated signaling pathway in trans-men | – Increases in splenic Tregs and inhibition of the immune response | (27) |
| LTA | Lymphotoxin Alpha | Trans-Men | – Upregulated the cytokine-mediated signaling pathway in trans-men | – Expressed on Tregs to modulate lymphatic endothelial cells | (28) |
| IL32 | Interleukin 32 | Cis-Women | – Upregulated the cytokine-mediated signaling pathway in trans-men | – Promotes Foxp3+ Treg Cell Development | (29) |
| CD83 | CD83 Molecule | Cis-Men | – Downregulated the cell activation/ growth pathways in trans-women | – Essential for Treg cell differentiation and stability | (30) |
| BCL6 | B-cell lymphoma 6 | Cis-Men | – Downregulated the cell activation/ growth pathways in trans-women | – Preserves the suppressive function of Tregs during Tumorigenesis | (31) |
| NR4A3 | Nuclear Receptor Subfamily 4 Group A Member 3 | Cis-Men | – Downregulated the cell activation/ growth pathways in trans-women | – Essential for thymic Treg development and immune homeostasis<br>– Directly controls a genetic program indispensable for Treg maintenance, function, and immune suppression<br>– Regulates T-cell tolerance to suppress autoimmunity via Tregs<br>– Promotes Treg differentiation | (14-17, 32) |
| CXCR4 | C-X-C Motif Chemokine Receptor 4 | Cis-Men | – Downregulated the cell activation/ growth pathways in trans-women | – Reverts the suppressive activity of Tregs in renal cancer | (33) |

| | | | | | |
|---|---|---|---|---|---|
| ZFP36L2 | ZFP36 Ring Finger Protein Like 2 | Cis-Men | – Downregulated the cell activation/ growth pathways in trans-women | – Suppresses the Function of Regulatory T Cells | (34) |
| SOCS2 | Suppressor of cytokine signaling 2 | Trans-Women | – Downregulated the cell activation/ growth pathways in trans-women | – Regulates Foxp3+ inducible Treg stability | (35) |
| SLC25A29 | Solute carrier family 25 member 29 | Cis-Women<br>Trans-Women | – Genes altered in all gender-unique comparisons<br>– Top 10 genes upregulated in cis-women (non-X/Y) | - | - |
| TIAM2 | TIAM Rac1 associated GEF 2 | Cis-Women<br>Trans-Women | – The top ranked genes clustering/separating gender-unique groups by sPLS-DA<br>– Top 10 genes upregulated in cis-women (non-X/Y) | - | - |
| MZF1-AS1 | MZF1 antisense RNA 1 | Cis-Men | – The top ranked genes clustering/separating gender-unique groups by sPLS-DA<br>– Top 10 genes upregulated in cis-men (non-X/Y) | - | - |
| CCR5 | C-C motif chemokine receptor 5 | Cis-Women<br>Trans-Women | – Genes associated with sex/gender and SLE by open target analysis<br>– Top 10 genes upregulated in cis-women (non-X/Y) | – Augments Treg differentiation and migration to sites of inflammation<br>– Mediates Treg migration to the tumor microenvironment | (36, 37) |
| TIMD4 | T cell immunoglobulin and mucin domain containing 4 | Cis-Women | – Genes associated with sex/gender and SLE by open target analysis<br>– Top 10 genes upregulated in cis-women (non-X/Y) | – Induces Tregs in gliomas to promote tumour tolerance | (38) |
| DTX1 | Deltex E3 ubiquitin ligase 1 | Cis-Men<br>Trans-Men | – Genes associated with sex/gender and SLE by open target analysis<br>– Top 10 genes upregulated in cis-men (non-X/Y) | – Sustains the stability and suppressive activity of regulatory T cells *in vivo* | (39) |
| BRCA2 | Breast cancer gene 2 | Cis-Women | – Genes associated with sex/gender and SLE by open target analysis<br>– Top 10 genes upregulated in cis-women (non-X/Y) | – Greater Treg infiltration to BRCA2 mutant breast cancers | (40) |
| KDM6A | Lysine demethylase 6A | Cis-Women | – Genes associated with sex/gender and SLE by open target analysis | - | - |
| MC1R | Melanocortin 1 receptor | Cis-Men | – Genes associated with sex/gender and SLE by open target analysis | - | - |

**Supplemental Table 4: Summary of important genes highlighted by this study and their role in Tregs**
Genes of interest highlighted by this study are displayed with their protein name, relevance to this study and their established role in Tregs from previous studies. Overlapping annotations from this study are highlighted by colour.

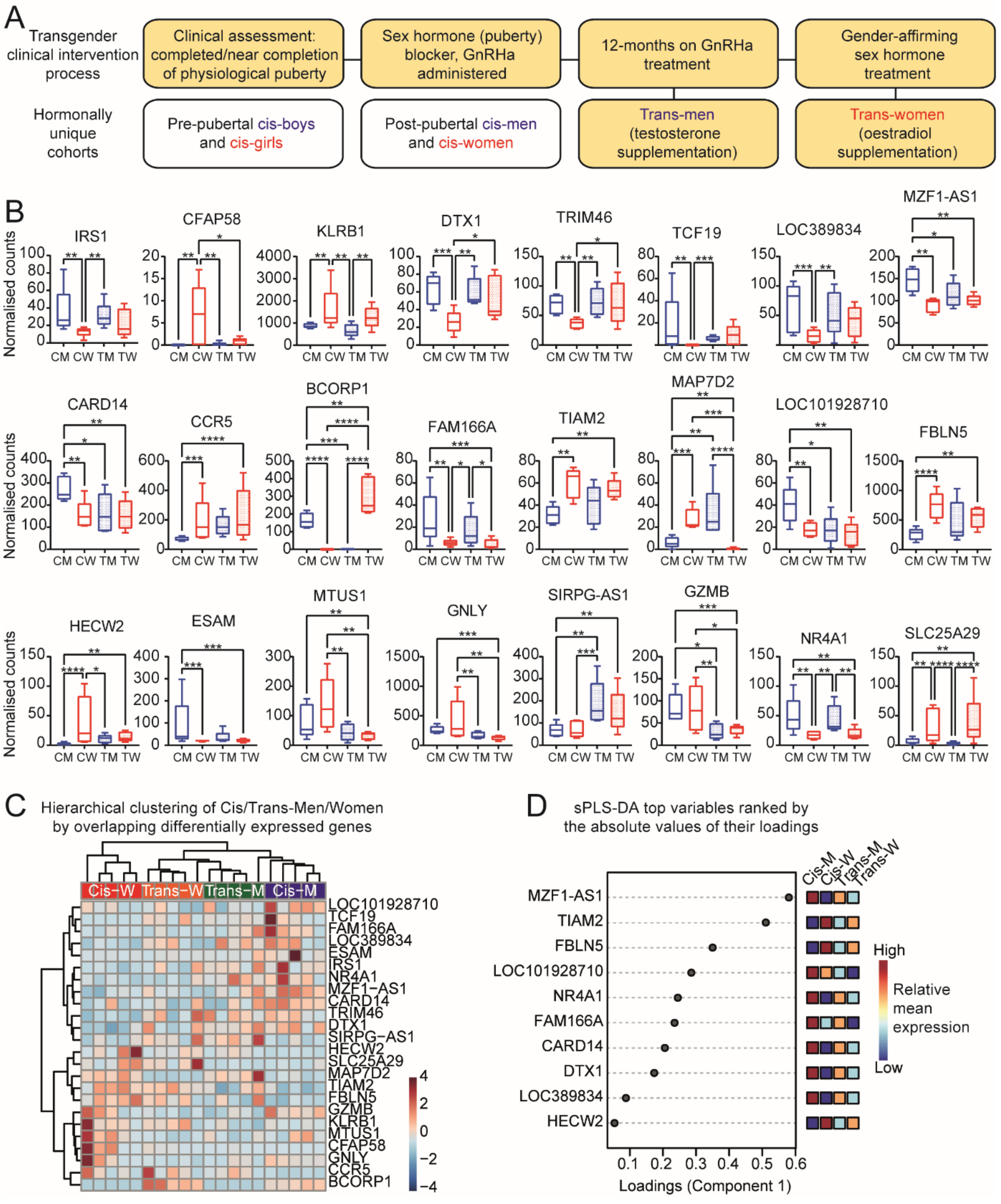


**Supplemental Figure 6: Overlapping differentially expressed Treg genes from different gender comparisons**

**(A)** Graphical summary of the gender-unique cohorts used in this analysis, including the transgender clinical process (GnRHa: gonadotropin-releasing hormone agonist). See Supplemental Methods for cohort details and gender-specific terminology descriptions. **(B)** Box and whisker plots displaying Treg gene expression by normalised counts of DEGs that overlapped between gender comparisons [cis-men (CM), cis-women (CW), trans-men (TM), and trans-women (TW)] from Figure 4A. Mean+SE. One-way ANOVA, **=p<0.01, ***=p<0.001, ****=p<0.0001. Numerical p values can be found in the Supplemental Data Table. **(C)** Hierarchical clustering heatmap (Clustvis, Pearson's) of normalised gene counts of of DEGs that overlapped between gender comparisons from Figure 4A. **(D)** Plot displaying the loading values and relative mean expression for top ranked genes for component 1 from the sPLS-DA analysis in Figure 4C.

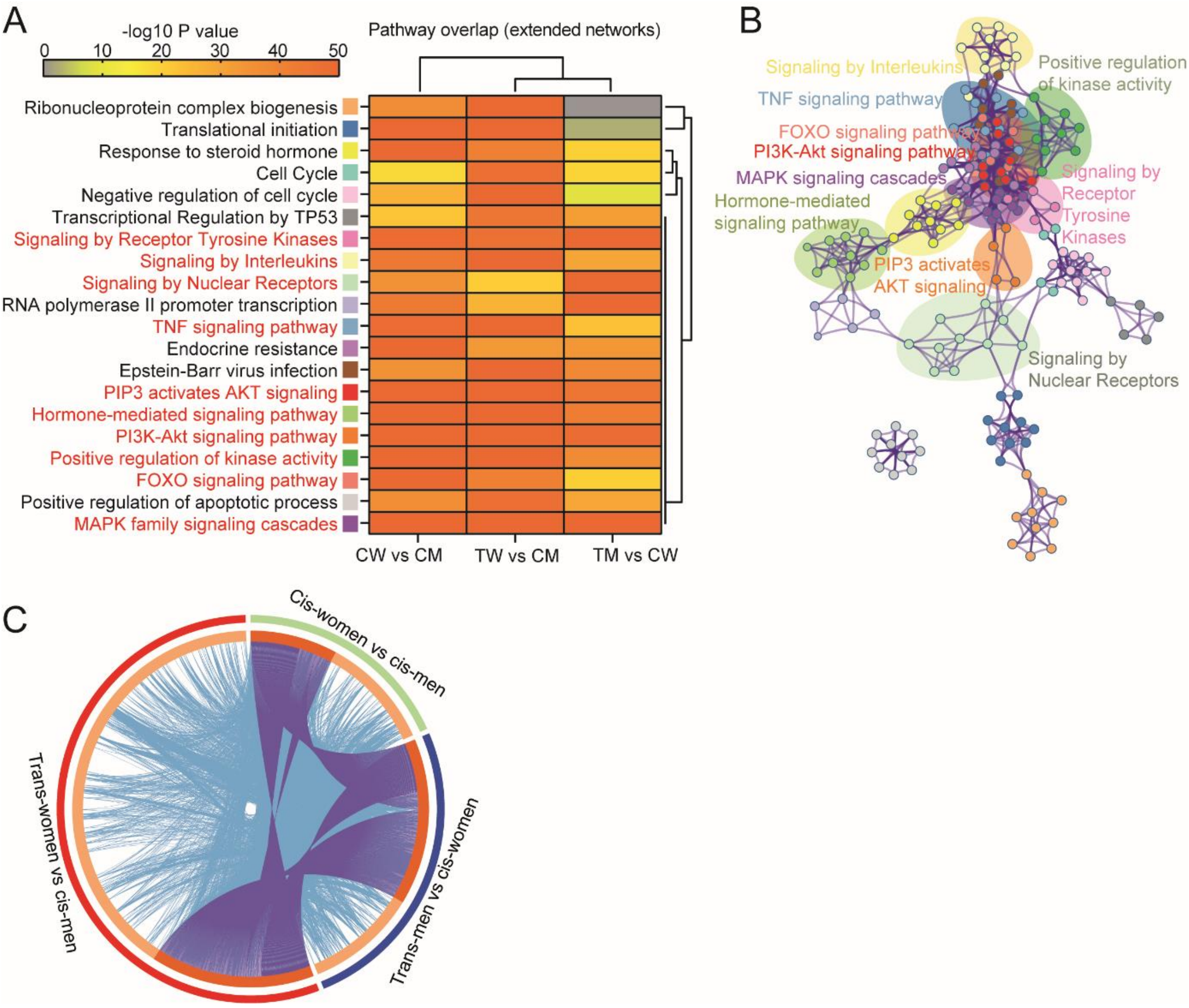


**Supplemental Figure 7: Overlapping genes and pathways between gender comparisons.**
**(A)** Clustered heatmap displaying -log10 p values of statistically significantly enriched genetic pathway ontology terms that overlap between extended protein-protein interaction network gene lists generated from respective DEG lists ($p<0.01$) identified in each gender comparison from (Figure 4A). Pathways involving secondary messenger signalling are highlighted in red. **(B)** Network diagram illustrating statistically significantly enriched genetic pathway ontology terms from comparisons from (B), matched colour labels. Similar terms with a high degree of redundancy were clustered into groups as depicted. Each node represents a statistically significantly enriched term, with node size proportional to the number of input genes annotated with this term. **(C)** Circos plot (Metascape) showing how DEGs from the input gene lists (first-order protein-protein interaction extended network gene lists) overlap between the different gender comparisons (A-B). On the outside, each arc colour represents the identity of each gene list. On the inside, dark orange colour represents the genes that appear in multiple lists and light orange colour represents genes that are unique to that gene list. Purple lines link the same gene that are shared by multiple gene lists. Blue lines link the different genes where they fall into the same ontology term (the term has to be statistically significantly enriched and with size no larger than 100).

| Treg phenotype: | Gender bias | Role of sex chromosomes vs sex hormones | Observation in JSLE |
|---|---|---|---|
| Circulating frequency/count | Men | Sex chromosomes and hormones | Differences absent (frequency increased in women only) |
| Suppressive capacity *in vitro* | Men | Sex chromosomes and hormones | Opposite (higher in women, suppression lowers in men) |
| Proportion of upregulated genes | Men | Sex chromosomes and hormones | Opposite (more in women) |
| Secondary messenger signalling pathways | Men | Sex hormones | Differences absent (alternative pathways are observed) |
| Cell growth/activation pathways | Men | Sex hormones | Differences absent (alternative pathways are observed) |
| Cytokine signalling/secretion pathways | Men | Sex hormones | Differences absent (alternative pathways are observed) |
| Gene proportion upregulated on the X/Y chromosome | Women | Sex chromosomes | Sex chromosome located genes maintain significance |

**Supplemental Table 5: Summary of sex and gender differences in Tregs from this study**

| Treg frequency, mean (SD) | On treatment | Off treatment | P value |
|---|---|---|---|
| Hydroxychloroquine (31/35 on treatment) | 6.42 (2.02) | 7.63 (1.55) | 0.26 |
| Mycophenolate mofetil (18/35 on treatment) | 6.87 (1.83) | 6.23 (2.16) | 0.35 |
| Prednisolone (17/35 on treatment) | 6.38 (2.20) | 6.73 (1.82) | 0.62 |
| Vitamin D (8/35 on treatment) | 5.58 (2.05) | 6.85 (1.92) | 0.11 |
| Methotrexate (3/35 on treatment) | 8.52 (1.19) | 6.38 (1.96) | 0.07 |
| Azathioprine (7/35 on treatment) | 6.72 (1.88) | 6.52 (2.05) | 0.81 |

**Supplemental Table 6: Comparison of Treg (CD4+, CD25+, CD127-) frequency between JSLE patients on and off specific treatments in the full cohort**
JSLE patients, n=35. Unpaired t test.

| | r value | P value |
|---|---|---|
| Systemic Lupus Erythematosus Disease Activity Index (SLEDAI) | 0.32 | 0.26 |
| Erythrocyte Sedimentation Rate (ESR) | -0.06 | 0.89 |
| Double-stranded DNA (dsDNA) titre | -0.22 | 0.62 |
| C3 | -0.18 | 0.74 |
| Lymphocyte count | -0.32 | 0.26 |

**Supplemental Table 7: Correlations between Treg (CD4+, CD25+, CD127-) frequency and clinical measures of JSLE disease activity**
JSLE patients, n=35. Spearman's correlation test.

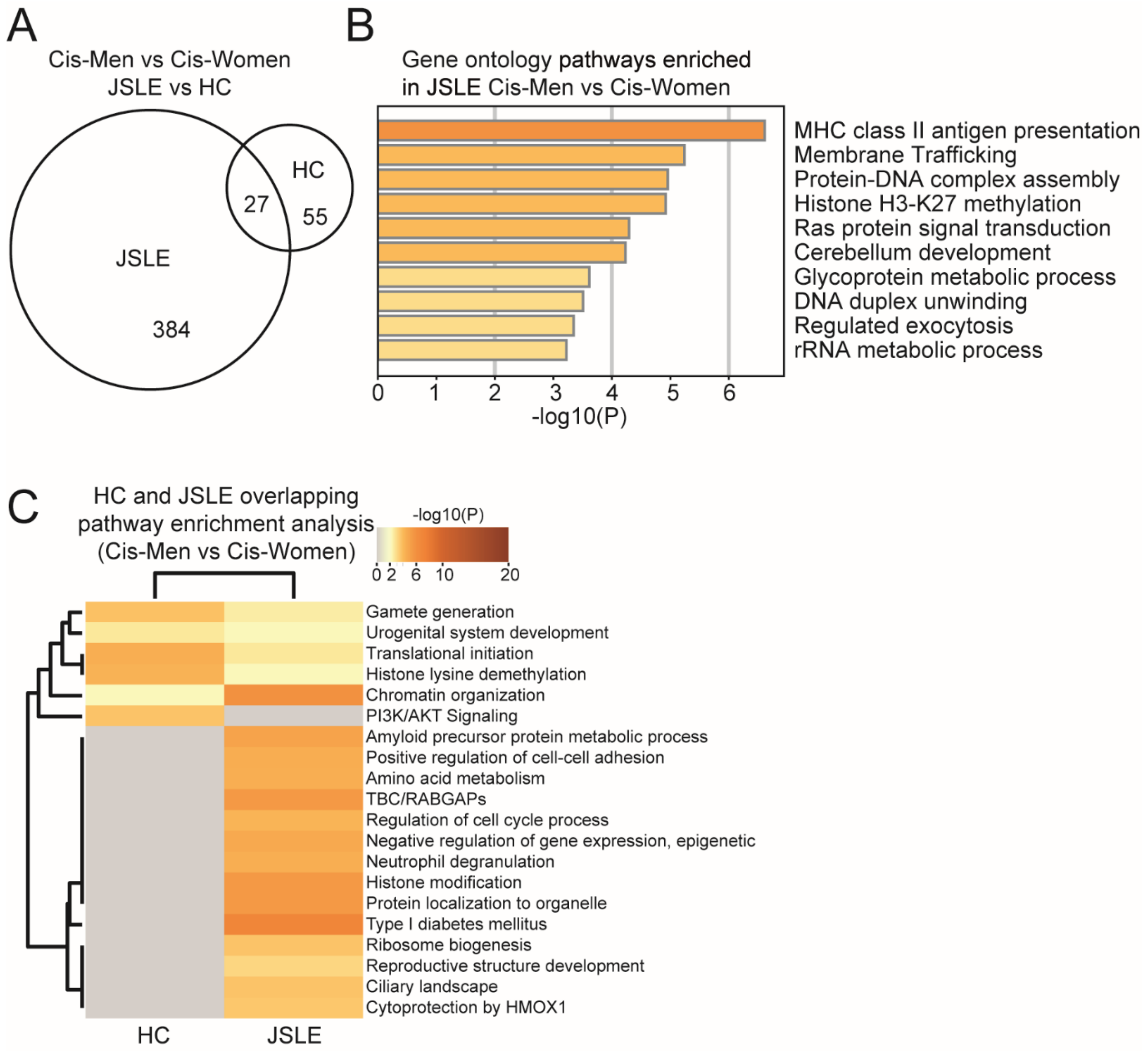


**Supplemental Figure 8: Sex differences in enriched gene ontology pathways between matched JSLE patients and HCs.**
**(A)** Venn analysis of overlapping genes from DEG comparisons (P<0.01) between healthy cis-men and cis-women (Figure 2A) vs JSLE cis-men and cis-women (Figure 5C). **(B)** Pathway analysis bar charts displaying the cluster significance -log10 p values of enriched pathway ontology terms from Treg DEGs (n=384) unique to the JSLE men vs women comparison in (A). **(C)** Clustered heatmap displaying -log10 p values of statistically significantly enriched genetic pathway ontology terms that overlap from DEG comparisons (P<0.01) between healthy cis-men and cis-women (Figure 2A) vs JSLE cis-men and cis-women (Figure 5C).

## Supplemental references


1. Hochberg MC. Updating the American College of Rheumatology revised criteria for the classification of systemic lupus erythematosus. Arthritis and Rheumatism. 1997;40(9):1725-.
2. Petri M, Orbai AM, Alarcon GS, Gordon C, Merrill JT, Fortin PR, et al. Derivation and validation of the systemic lupus international collaborating clinics classification criteria for systemic lupus erythematosus. Arthritis and Rheumatism. 2012;64(8):2677-86.
3. Robinson GA, Peng J, Dönnes P, Coelewij L, Naja M, Radziszewska A, et al. Disease-associated and patient-specific immune cell signatures in juvenile-onset systemic lupus erythematosus: patient stratification using a machine-learning approach. Lancet Rheumatol. 2020;2(8):e485-e96.
4. R Core Team. R: A language and environment for statistical computing. Available online at https://www.R-project.org/ Vienna, Austria: R Foundation for Statistical Computing; 2018 [
5. Gentleman RC, Carey VJ, Bates DM, Bolstad B, Dettling M, Dudoit S, et al. Bioconductor: open software development for computational biology and bioinformatics. Genome Biology. 2004;5(10).
6. Anders S, Huber W. Differential expression analysis for sequence count data. Genome Biology. 2010;11(10).
7. Love MI, Huber W, Anders S. Moderated estimation of fold change and dispersion for RNA-seq data with DESeq2. Genome Biology. 2014;15(12).
8. Szklarczyk D, Franceschini A, Wyder S, Forslund K, Heller D, Huerta-Cepas J, et al. STRING v10: protein-protein interaction networks, integrated over the tree of life. Nucleic Acids Research. 2015;43(D1):D447-D52.
9. Andy Liaw MW. Classification and regression by randomForest. R News: R News; 2002. p. 18-22.
10. Ho TK. Random decision forests. Proceedings of the Third International Conference on Document Analysis and Recognition (Volume 1) - Volume 1. 844681: IEEE Computer Society; 1995. p. 278.
11. Choi MY, Ma C. Making a big impact with small datasets using machine-learning approaches. The Lancet Rheumatology. 2020;2(8):e451-e2.
12. Robinson GA, Peng J, Dönnes P, Coelewij L, Naja M, Radziszewska A, et al. Disease-associated and patient-specific immune cell signatures in juvenile-onset systemic lupus erythematosus: patient stratification using a machine-learning approach. The Lancet Rheumatology. 2020;2(8):e485-e96.
13. Kuhn M, editor The caret Package2007.
14. Hiwa R, Nielsen HV, Mueller JL, Mandla R, Zikherman J. NR4A family members regulate T cell tolerance to preserve immune homeostasis and suppress autoimmunity. JCI Insight. 2021;6(17).
15. Hibino S, Chikuma S, Kondo T, Ito M, Nakatsukasa H, Omata-Mise S, et al. Inhibition of Nr4a Receptors Enhances Antitumor Immunity by Breaking Treg-Mediated Immune Tolerance. Cancer Res. 2018;78(11):3027-40.
16. Sekiya T, Kashiwagi I, Yoshida R, Fukaya T, Morita R, Kimura A, et al. Nr4a receptors are essential for thymic regulatory T cell development and immune homeostasis. Nat Immunol. 2013;14(3):230-7.
17. Sekiya T, Kondo T, Shichita T, Morita R, Ichinose H, Yoshimura A. Suppression of Th2 and Tfh immune reactions by Nr4a receptors in mature T reg cells. J Exp Med. 2015;212(10):1623-40.
18. Polanczyk MJ, Hopke C, Vandenbark AA, Offner H. Treg suppressive activity involves estrogen-dependent expression of programmed death-1 (PD-1). International Immunology. 2007;19(3):337-43.
19. Polanczyk MJ, Hopke C, Huan JY, Vandenbark AA, Offner H. Enhanced FoxP3 expression and Treg cell function in pregnant and estrogen-treated mice. Journal of Neuroimmunology. 2005;170(1-2):85-92.
20. Polanczyk MJ, Carson BD, Subramanian S, Afentoulis M, Vandenbark AA, Ziegler SF, et al. Cutting edge: Estrogen drives expansion of the CD4(+) CD25(+) regulatory T cell compartment. Journal of Immunology. 2004;173(4):2227-30.
21. Lo Re S, Lecocq M, Uwambayinema F, Yakoub Y, Delos M, Demoulin JB, et al. Platelet-derived growth factor-producing CD4+ Foxp3+ regulatory T lymphocytes promote lung fibrosis. Am J Respir Crit Care Med. 2011;184(11):1270-81.
22. Crellin NK, Garcia RV, Hadisfar O, Allan SE, Steiner TS, Levings MK. Human CD4+ T cells express TLR5 and its ligand flagellin enhances the suppressive capacity and expression of FOXP3 in CD4+CD25+ T regulatory cells. J Immunol. 2005;175(12):8051-9.
23. van Maren WW, Jacobs JF, de Vries IJ, Nierkens S, Adema GJ. Toll-like receptor signalling on Tregs: to suppress or not to suppress? Immunology. 2008;124(4):445-52.
24. Kabelitz D. Expression and function of Toll-like receptors in T lymphocytes. Curr Opin Immunol. 2007;19(1):39-45.
25. Park SH, Ham S, Lee A, Möller A, Kim TS. NLRP3 negatively regulates Treg differentiation through Kpna2-mediated nuclear translocation. J Biol Chem. 2019;294(47):17951-61.
26. Wang H, Franco F, Tsui YC, Xie X, Trefny MP, Zappasodi R, et al. CD36-mediated metabolic adaptation supports regulatory T cell survival and function in tumors. Nat Immunol. 2020;21(3):298-308.

27. Prescott D, Maisonneuve C, Yadav J, Rubino SJ, Girardin SE, Philpott DJ. NOD2 modulates immune tolerance via the GM-CSF-dependent generation of CD103. Proc Natl Acad Sci U S A. 2020;117(20):10946-57.
28. Piao W, Xiong Y, Li L, Saxena V, Smith KD, Hippen KL, et al. Regulatory T Cells Condition Lymphatic Endothelia for Enhanced Transendothelial Migration. Cell Rep. 2020;30(4):1052-62.e5.
29. Han L, Chen S, Chen Z, Zhou B, Zheng Y, Shen L. Interleukin 32 Promotes Foxp3 + Treg Cell Development and CD8 + T Cell Function in Human Esophageal Squamous Cell Carcinoma Microenvironment. Front Cell Dev Biol. 2021;9:704853.
30. Doebbeler M, Koenig C, Krzyzak L, Seitz C, Wild A, Ulas T, et al. CD83 expression is essential for Treg cell differentiation and stability. JCI Insight. 2018;3(11).
31. Li Y, Wang Z, Lin H, Wang L, Chen X, Liu Q, et al. Bcl6 Preserves the Suppressive Function of Regulatory T Cells During Tumorigenesis. Front Immunol. 2020;11:806.
32. Jennings E, Elliot TAE, Thawait N, Kanabar S, Yam-Puc JC, Ono M, et al. Nr4a1 and Nr4a3 Reporter Mice Are Differentially Sensitive to T Cell Receptor Signal Strength and Duration. Cell Rep. 2020;33(5):108328.
33. Santagata S, Napolitano M, D'Alterio C, Desicato S, Maro SD, Marinelli L, et al. Targeting CXCR4 reverts the suppressive activity of T-regulatory cells in renal cancer. Oncotarget. 2017;8(44):77110-20.
34. Makita S, Takatori H, Iwata A, Tanaka S, Furuta S, Ikeda K, et al. RNA-Binding Protein ZFP36L2 Downregulates Helios Expression and Suppresses the Function of Regulatory T Cells. Front Immunol. 2020;11:1291.
35. Knosp CA, Schiering C, Spence S, Carroll HP, Nel HJ, Osbourn M, et al. Regulation of Foxp3+ inducible regulatory T cell stability by SOCS2. J Immunol. 2013;190(7):3235-45.
36. de Oliveira CE, Gasparoto TH, Pinheiro CR, Amôr NG, Nogueira MRS, Kaneno R, et al. CCR5-Dependent Homing of T Regulatory Cells to the Tumor Microenvironment Contributes to Skin Squamous Cell Carcinoma Development. Mol Cancer Ther. 2017;16(12):2871-80.
37. Jiao X, Nawab O, Patel T, Kossenkov AV, Halama N, Jaeger D, et al. Recent Advances Targeting CCR5 for Cancer and Its Role in Immuno-Oncology. Cancer Res. 2019;79(19):4801-7.
38. Xu L, Xiao H, Xu M, Zhou C, Yi L, Liang H. Glioma-derived T cell immunoglobulin- and mucin domain-containing molecule-4 (TIM4) contributes to tumor tolerance. J Biol Chem. 2011;286(42):36694-9.
39. Hsiao HW, Hsu TS, Liu WH, Hsieh WC, Chou TF, Wu YJ, et al. Deltex1 antagonizes HIF-1α and sustains the stability of regulatory T cells in vivo. Nat Commun. 2015;6:6353.
40. Li Y, Dong W, Zhang P, Zhang T, Ma L, Qu M, et al. Comprehensive Analysis of Regulatory Factors and Immune-Associated Patterns to Decipher Common and *BRCA1/2* Mutation-Type-Specific Critical Regulation in Breast Cancer. Front Cell Dev Biol. 2021;9:750897.
